# Diet and feeding pattern modulate diurnal dynamics of the ileal microbiome and transcriptome

**DOI:** 10.1016/j.celrep.2022.111008

**Published:** 2022-07-05

**Authors:** Ana Carolina Dantas Machado, Steven D. Brown, Amulya Lingaraju, Vignesh Sivaganesh, Cameron Martino, Amandine Chaix, Peng Zhao, Antonio F.M. Pinto, Max W. Chang, R. Alexander Richter, Alan Saghatelian, Alan R. Saltiel, Rob Knight, Satchidananda Panda, Amir Zarrinpar

**Affiliations:** 1 Division of Gastroenterology, University of California, San Diego, 9500 Gilman Drive, MC 0983, La Jolla, CA, USA; 2 Department of Medicine, University of California, San Diego, 9500 Gilman Drive, MC 0983, La Jolla, CA, USA; 3 Department of Pediatrics, School of Medicine, University of California, San Diego, La Jolla, CA, USA; 4 Bioinformatics and Systems Biology Program, University of California, San Diego, La Jolla, CA, USA; 5 Center for Microbiome Innovation, University of California, San Diego, 9500 Gilman Drive, MC 0983, La Jolla, CA, USA; 6 Department of Nutrition and Integrative Physiology, University of Utah, Salt Lake City, UT, USA; 7 Division of Metabolism and Endocrinology, Department of Medicine, University of California, San Diego, La Jolla, CA, USA; 8 Clayton Foundation Laboratories for Peptide Biology, the Salk Institute for Biological Studies, La Jolla, CA, USA; 9 Department of Pharmacology, Department of Medicine, University of California, San Diego, La Jolla, CA, USA; 10 Institute of Diabetes and Metabolic Health, University of California, San Diego, 9500 Gilman Drive, MC 0983, La Jolla, CA, USA; 11 Department of Computer Science and Engineering, University of California, San Diego, La Jolla, CA, USA; 12 Department of Bioengineering, University of California, San Diego, La Jolla, CA, USA; 13 Regulatory Biology Laboratory, The Salk Institute, 10010 N. Torrey Pines Road, La Jolla, CA, USA; 14 VA Health Sciences, San Diego, La Jolla, CA, USA; 15 Present address: Department of Biochemistry and Structural Biology, University of Texas Health Science Center at San Antonio. Mays Cancer Center, University of Texas Health Science Center at San Antonio.; 16 Lead contact

## Abstract

Compositional oscillations of the gut microbiome are essential for normal peripheral circadian rhythms, both of which are disrupted in diet-induced obesity (DIO). Although time-restricted feeding (TRF) maintains circadian synchrony and protects against DIO, its impact on the dynamics of the cecal gut microbiome is modest. Thus, other regions of the gut, particularly the ileum, the nexus for incretin and bile acid signaling, may play an important role in entraining peripheral circadian rhythms. We demonstrate the effect of diet and feeding rhythms on the ileal microbiome composition and transcriptome in mice. The dynamic rhythms of ileal microbiome composition and transcriptome are dampened in DIO. TRF partially restores diurnal rhythms of the ileal microbiome and transcriptome, increases GLP-1 release, and alters the ileal bile acid pool and farnesoid X receptor (FXR) signaling, which could explain how TRF exerts its metabolic benefits. Finally, we provide a web resource for exploration of ileal microbiome and transcriptome circadian data.

## In brief

Dantas Machado et al. show that time-restricted feeding restores diurnal dynamics of the ileal microbiome and transcriptome that are lost under high-fat diet *ad libitum* food access. Time restricted feeding restores ileal microbiome oscillations, circadian genes, bile acid signaling, and GLP-1 regulation.

## Graphical Abstract

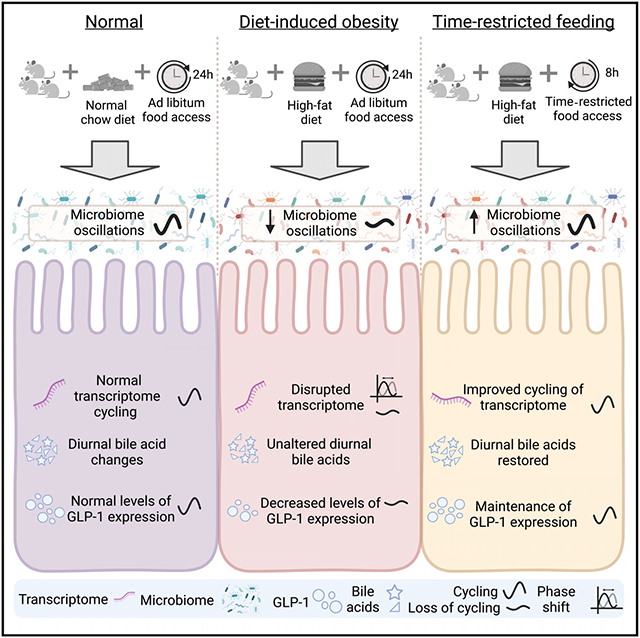

## INTRODUCTION

The gut microbiome plays a crucial role in many physiological processes such as digestion and nutrient absorption, vitamin synthesis, and immune system development and programming (Gilbert et al., 2018; Lynch and Pedersen, 2016; Sharon et al., 2014). Recent studies show an added role for the gut microbiome: the entrainment of intestinal and hepatic circadian rhythms (Alvarez et al., 2020; Bishehsari et al., 2020; Frazier and Chang, 2019; Thaiss et al., 2016; Zarrinpar et al., 2016). Diet and feeding/fasting cycles drive diurnal oscillations of microbial communities and secondary metabolites in the gut luminal environment. These oscillations are necessary for the entrainment of peripheral circadian clocks and with it the diurnal expression of hepatic and intestinal metabolic regulators that control glucose, cholesterol, and fatty acid homeostasis and overall host metabolic health (Bishehsari et al., 2020; Frazier and Chang, 2019; Thaiss et al., 2014; Zarrinpar et al., 2016). However, studies that investigate the relationship between the gut microbiome and peripheral clocks using microbiome-depletion models (Leone et al., 2015; Mukherji et al., 2013; Wang et al., 2017; Weger et al., 2019) do not explain whether diet-induced perturbations in microbiome dynamics could influence host metabolism through circadian dyssynchrony (Saran et al., 2020; Zarrinpar et al., 2016).

Diet-induced obesity (DIO) may give additional insight into the relationship between perturbed luminal diurnal dynamics and host metabolism. Conventionally raised mice given *ad libitum* access to high-fat diet (HFD) lose their diurnal feeding pattern and consume twice as many calories in the light period as they do before their diet was switched (Kohsaka et al., 2007). This disruption of feeding pattern leads to dampening of hepatic circadian rhythms and dysregulation of metabolic regulators that are associated with increased adiposity, ectopic steatosis, and insulin resistance (Chaix et al., 2014; Hatori et al., 2012). Even though DIO disrupts the normal cyclical fluctuation of the gut microbiome (Leone et al., 2015; Zarrinpar et al., 2014), the impact of this hallmark characteristic on intestinal circadian rhythms is poorly understood.

Time-restricted feeding (TRF), wherein food consumption is consolidated to a window of 8–10 h during the dark (the active period in mice), has numerous benefits on host metabolic health (Chaix et al., 2014, 2019a, 2021; Hatori et al., 2012; Zarrinpar et al., 2014). In mice consuming HFD, TRF results in reduced adiposity, decreased inflammation, improved glucose tolerance and cholesterol homeostasis, and reversal of pre-existing metabolic syndrome, regardless of the type of obesogenic diet fed (Chaix et al., 2014; Hatori et al., 2012; Sherman et al., 2012; Zarrinpar et al., 2014). However, despite many metabolic improvements, the effects of TRF on the cyclical fluctuations of the cecal microbiota were modest. However, its effects on serum and fecal secondary metabolites were dramatic and could potentially explain how TRF entrains peripheral clock or imparts its benefits (Chaix et al., 2014; Zarrinpar et al., 2014).

Most gut microbiome research is focused on the large intestine or its more accessible surrogate, stool (Gu et al., 2013; Martinez-Guryn et al., 2019; Thaiss et al., 2014, 2016). However, other regions of the gut play a far more important role in host metabolic homeostasis (Tuganbaev et al., 2020). In particular, the ileum is unique in its functions of digestion and absorption as well as in its microbial composition (Gu et al., 2013; Martinez-Guryn et al., 2019). Ileal microbes play a vital role in metabolic processes and immune modulation, both within the intestinal microenvironment and systemically. For example, bile acids are important signaling molecules in the gut, and bile acid signaling receptors such as the farnesoid X receptor (FXR) and the G protein-coupled bile acid receptor 1 (TGR5), which regulate several metabolic pathways, are highly expressed in the ileum (Ridlon et al., 2016; Wahlstrom et al., 2016). FXR activation in the ileum results in the upregulation of protective, anti-bacterial immune responses (Inagaki et al., 2006). Regulation of glucose metabolism via GLP-1 signaling also occurs primarily in the ileum (Paternoster and Falasca, 2018), with the microbiome being an important component for the diurnal regulation of incretin release (Martchenko et al., 2020). Furthermore, diurnal rhythmicity of the small intestinal microbiome, shaped by diurnal feeding patterns, is critical to maintaining gut barrier function and immune homeostasis (Tuganbaev et al., 2020).

Despite this, there are few studies that highlight the importance of the ileal gut microbiome and its impact on host metabolic health. In this study, we investigated the diurnal dynamics of the microbiome composition and transcriptome in the ileum under normal and HFD feeding conditions. Our goal was to determine how peripheral circadian rhythm is entrained in the ileum and to characterize the role of the microbiome and transcriptome under normal feeding, HFDs, and TRF given the importance of incretin release and bile acid reabsorption signaling in the ileum. The ileum has highly cyclical luminal microbial dynamics and host transcriptomics that are greatly perturbed with DIO. TRF has a large impact on the ileal microbiome composition, imposing a strong diurnal dynamic that is distinct from both DIO mice and mice on a normal chow diet (NCD). Moreover, TRF prevents the loss of host ileal diurnal transcriptome dynamics and maintains the rhythmicity of the intestinal clock. These effects on ileal dynamics could underlie TRF metabolic benefits and effects on peripheral clock entrainment.

## RESULTS

### Diet and feeding pattern affect composition and diurnal dynamics of ileal gut microbiome

**Figure 1.**
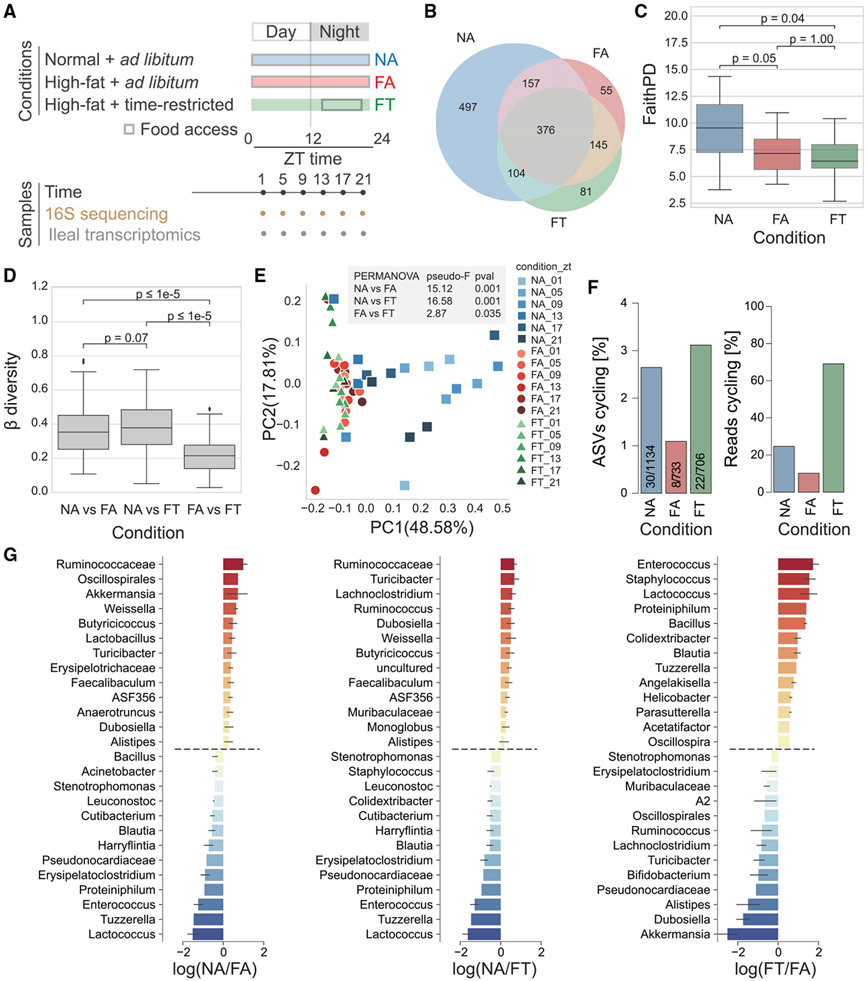
Microbiota diversity and cyclical pattern at the ileum (A) Schematic representation of study design and sample collection. (B) Shared ASVs between conditions. (C) Measures of α-diversity within each sample based on phylogenetic distance (Faith’s PD). (D) Overall β-diversity across conditions time points. (E) Principal coordinate analysis (PCoA) of ASVs colored by condition and collection time. (F) Cycling (JTK_CYCLE algorithm, MetaCycle) based on the total number of ASVs or reads. (G) Differential ranking between conditions as determined by Songbird. Statistical significance was assessed with the Mann-Whitney U test. *p < 0.05; **p < 0.01; ***p < 0.001; n.s., not significant. Three mice per time point were used for each condition, for a total of NA (n = 18), FA (n = 18), FT (n = 18).

To investigate the effects of TRF on host ileal cyclical activity, we collected ileal samples from the same mice as described in a previous TRF experiment where the cecal microbiome was characterized (Zarrinpar et al., 2014). The phenotype of mice with time-restricted access to HFD (FT) was compared with mice with *ad libitum* access to HFD (FA; which is the same as the DIO model) and mice with *ad libitum* access to NCD (NA; control mice) (Figure 1A). After 8 weeks under these conditions, whole ileum was collected every 4 h during a 24-h period (three mice per time point per feeding condition) to investigate diurnal dynamics of both luminal and adherent bacteria. TRF improves body weight and blood glucose levels associated with HFD (Figures S1A and S1B) despite isocaloric food intake compared with DIO (Chaix et al., 2014; Hatori et al., 2012; Sherman et al., 2012).

The composition of the ileal microbiome, as determined by 16S amplicon sequencing of the host sample, was shaped by diet (Figure 1B). Although mice in the NA condition had 1,134 amplicon sequence variants (ASVs) across all time points, mice fed an HFD had about two-thirds as many in both FA and FT feeding conditions (733 in FA; 706 in FT; Figure 1B). Although it may seem like there are regional differences with a higher number of ASVs in the ileum compared with the cecum (Figures 1B and S1C) (Zarrinpar et al., 2014), we cannot rule out that this may be due to differences in 16S amplicon sequencing techniques (see STAR Methods). HFD reduced ileal microbiome α-diversity, as determined by Faith’s phylogenetic diversity (PD) (p ≤ 0.05 for FA and FT; Figure 1C), although α-diversity measures that did not rely on phylogeny, such as Shannon index, were not changed between diet and feeding conditions (Figure S1D). In the cecum, the Faith’s PD α-diversity of the microbiome was decreased, although the Shannon index was unchanged between diet and feeding condition (Figure S1E). Measures of α-diversity (i.e., Faith’s PD) were not an indicator of improved metabolic health in the FT condition since in both cecum and ileum they were significantly lower than the NA condition.

To assess similarity of the microbiome between our three different conditions, we calculated weighted UniFrac distances to assess β-diversity. Between-class distances show that both HFD conditions FA and FT are significantly different from our NCD control mice in both cecum and ileum (Figures 1D and S1F). However, the β-diversity distances between our FA and FT mice were significantly lower, demonstrating that there is less difference in the microbiome composition of HFD animals and that diet has a larger influence on microbiome composition than feeding pattern. Principal coordinates analysis (PCoA) of microbiome samples showed similar results. The NA ileal microbiomes were distinct from both FA and FT ileal microbiomes (NA versus FA permutational multivariate analysis of variance [PERMANOVA] pseudo-F = 15.12, p = 0.001; NA versus FT PERMANOVA pseudo-F = 16.58, p = 0.001; Figure 1E). This stark difference was also true in the cecum (NA versus FA PERMANOVA pseudo-F = 13.38, p = 0.001; NA versus FT PERMANOVA pseudo-F = 14.87, p = 0.001; Figure S1G). However, feeding pattern created a distinction in the ileal microbiome composition of FA and FT mice as well (FA versus FT PERMANOVA pseudo-F = 2.87, p = 0.035; Figure 1E) as well as the cecal microbiome (FA versus FT PERMANOVA pseudo-F = 3.52848; p = 0.015; Figure S1G). Despite an apparent variation in diversity at zeitgeber time (ZT) 13 in the ileum, which is when TRF are given access to food, microbiome β-diversity dynamics between NCD and each HFD condition (FA or FT) are not significant in both ileum and cecum (Figure S1H).

Because the maintenance of microbial, circadian, and metabolic rhythms has been linked with the benefits of TRF on metabolic function (Saran et al., 2020; Zarrinpar et al., 2016), we next investigated microbiome cyclical patterns using the JTK_CYCLE algorithm (Hughes et al., 2010) in MetaCycle (Wu et al., 2016). Compared with the NA mice, FA mice had less than half the number of cycling ASVs (Figure 1F). However, TRF displays a similar level of cyclical fluctuations observed for animals under the NA condition despite being fed an HFD. This is likely a result of a few predominantly cyclical ASVs, as shown by the percentage of total ASV reads cycling (Figure 1F). The main taxa differentially ranked genera between NCD and HFD, as determined using Songbird (Morton et al., 2019b), include Ruminococcaceae, *Lactococcus*, *Tuzzerella*, and *Enterococcus*. On the other hand, the genera *Lactococcus* and *Erysipelatoclostridium* were identified as enriched in FA compared with FT (Figure 1G). Together, these findings indicate the ileal microbiome displays robust diurnal dynamics that are disturbed by diet in a feeding-pattern-dependent manner.

### TRF maintains bacterial cyclical dynamics in the ileum

**Figure 2.**
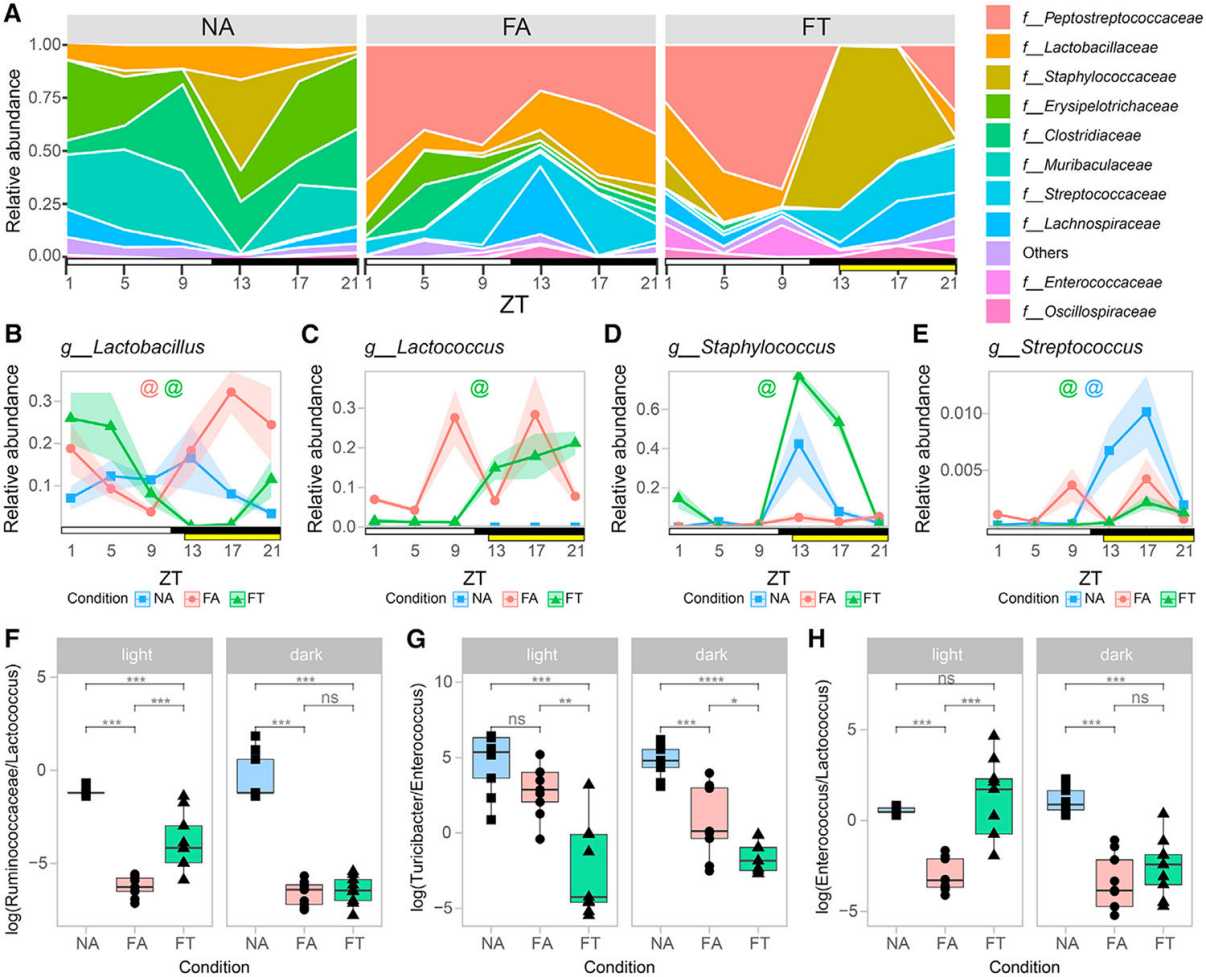
Microbiota cycling dynamics signatures of the ileum at the family and genus level (A) Relative abundance of top 10 bacterial families by time point for each condition. (B–E) Relative abundance for specific bacteria at the genus taxonomic level. Shaded areas show standard error of mean (SEM). (F–H) Log ratios of major differentially ranked bacteria (obtained from Songbird) separated by light and dark phases. Colored “@” symbols represent bacteria cycling under specific feeding conditions (NA = blue; FA = red; FT = green; MetaCycle JTK_CYCLE method p value <0.05). Statistical significance was assessed with Mann-Whitney U test. *p < 0.05; **p < 0.01; ***p < 0.001. Light and dark periods are represented by white and black horizontal bars, respectively. TRF food interval is represented by a yellow bar. Three mice per time point were used for each condition, for a total of NA (n = 18), FA (n = 18), FT (n = 18).

To characterize the cyclical oscillations of the ileal microbiome and determine their importance in host metabolism, we investigated the relative abundances of taxa under different diet and feeding conditions over time. Firmicutes were the predominant phylum across all conditions. Similar to what has been observed in the cecum and stool, the abundance of Bacteroidota decreased dramatically under both HFD conditions (Figure S2A). Family-level analysis revealed that diet and food access modulated oscillations in bacterial taxa and abundance over a 24-h period, as exemplified by the top 10 most representative families (Figure 2A). Bacteria at the highest and lowest differential rankings or with different rhythmicity between conditions (Figures 1G and S2B) were further analyzed. At the genus level, *Lactobacillus* was found in all conditions but had a cyclical pattern only under HFD conditions (Figure 2B), with its level greatly reduced during the dark phase in FT when the mice have access to food (Figure 2B). On the other hand, *Lactococcus*, which is found in the irradiated HFD (Bisanz et al., 2019), displayed abundance in agreement with food ingestion patterns of animals under both HFD conditions, although rhythmicity was observed only in FT (Figure 2C). During the beginning of the dark phase, a spike was seen in *Staphylococcus* in NA and FT mice, whereas the FA mice did not have this spike (Figures 2A and 2D). Despite this increase in both NA and FT mice, *Staphylococcus* had significant cycling in FT but not NA (Figure 2D). Finally, *Streptococcus* levels followed the feeding pattern of mice under the different conditions, and only displayed rhythmicity in NA and FT mice (Figure 2E).

Because log ratios of bacteria, as opposed to relative abundances, take advantage of reference frames and are not dependent on bacterial load information, they are more robust and yield more reproducible measures. Thus, we next evaluated if differences were observed in the log ratios of some high-ranking microbes between light and dark phases. Log ratios for bacteria whose differentials were predominant across conditions or between light and dark phases are shown in Figure S2C. Differences between conditions occurred during the light and dark cycles for Ruminococcaceae/*Lactococcus*, *Turicibacter/Enterococcus* and *Enterococcus/Lactococcus* (Figures 2F-2H). Together, our findings suggest that TRF affects the diurnal dynamics of the gut microbiome composition at the ileum. An interactive tool allows researchers to investigate bacterial families at https://zarrinparlab.github.io/ti_cycling_paper/ti_16S_family_abundance.html (Figure S3).

### Host clock genes influence the diurnal dynamics of ileal microbiome composition

**Figure 3.**
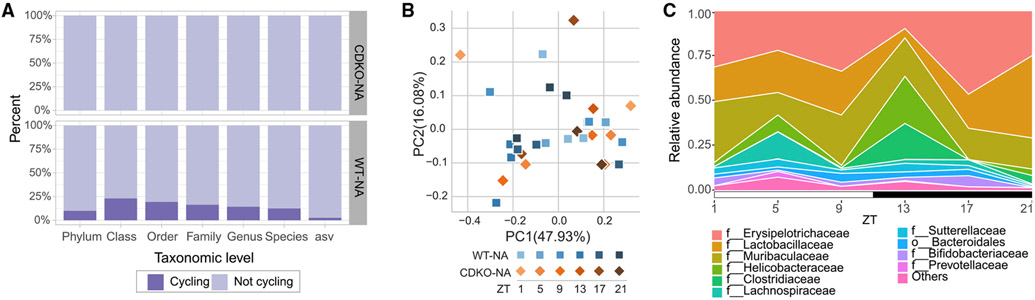
Ileal microbiota cycling and composition in CDKO mice (A) Bar plot illustrates percentage of microbes at various taxonomic levels displaying cyclical dynamics in *CDKO* mice compared with WT mice with *ad libitum* access to normal chow (NA). (B) PCoA of weighted UniFrac distances for CDKO-NA compared with WT-NA. (C) Relative abundance of top 10 families in *CDKO* mice by time point. Light and dark periods are represented by white and black horizontal bars, respectively. Three or two mice per time point were used for WT-NA (n = 18) and CDKO-NA (n = 12), respectively. Animals are from different maternal lines.

To test whether circadian clock genes are important for microbiome dynamics, we used a *Cry1;Cry2* double-knockout (CDKO) mouse (Vitaterna et al., 1999). Since HFD disrupts circadian gene expression, we used a CDKO mouse model with *ad libitum* access to an NCD (CDKO-NA) to investigate diurnal dynamics of the gut microbiome. Feeding behavior and metabolic phenotyping of these animals is well documented in previous studies, where CDKO-NA mice lose diurnal feeding pattern and have disrupted feeding patterns (Chaix et al., 2019a; Vollmers et al., 2009). Microbial cycling in the ileum was completely abolished in CDKO-NA mice (Figure 3A). PCoA analysis (Figure 3B, CDKO-NA versus wild-type [WT]-NA PERMANOVA pseudo-F = 3.0083, p = 0.045) reflects the perturbed rhythms in these mice (e.g., feeding, sleeping). The most prevalent bacteria found in the ileum of these mice were members of the Erysipelotrichaceae and Lactobacillaceae families (Figure 3C). These results further suggest that the host molecular circadian clock is linked to microbiome ileum dynamics and that interfering with the host circadian clock disrupts the diurnal rhythm of the ileal microbiome, likely through a disturbed feeding pattern.

### TRF maintains host transcriptome diurnal dynamics and intestinal clock

**Figure 4.**
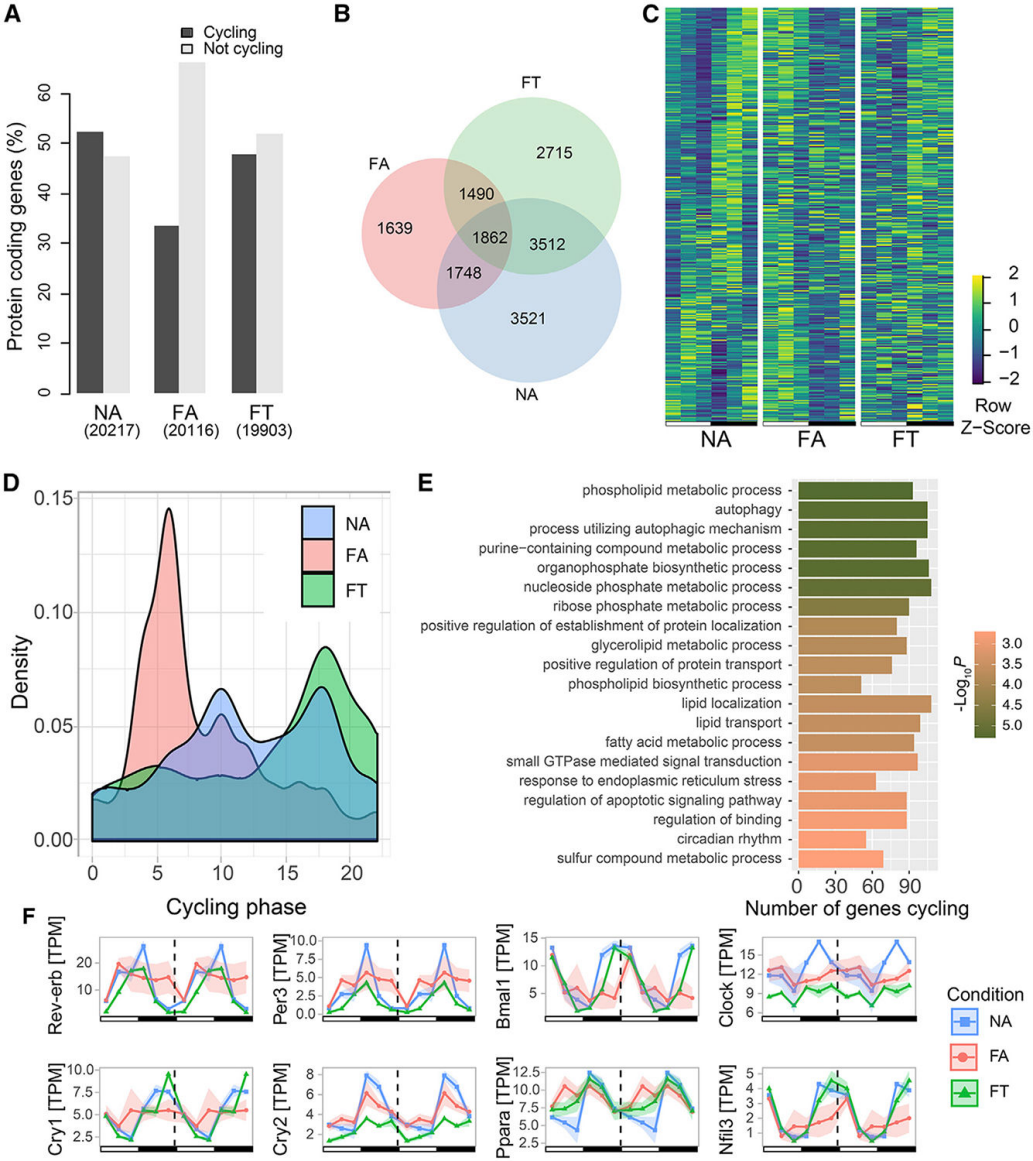
Host transcriptome cycling dynamics in the ileum (A) Percentage of cycling and non-cycling transcripts by condition (chi-squared p <0.05). (B) Venn diagram showing number of cycling protein-coding transcripts. (C) Heatmaps show the expression levels of the 1,862 genes that have circadian cycling in all three conditions. Rows were sorted by gene expression cycling phase based on NA. Values are *Z* scores of expression levels in transcripts per million (TPM). (D) Phase distribution of cycling genes. (E) Enriched gene ontology (GO) terms based on genes that lost cycling in FA, but not FT. (F) Double plot showing gene expression of circadian genes. Three mice per time point were used for each condition, for a total of NA (n = 18), FA (n = 18), FT (n = 18). Shading areas show standard error of mean (SEM).

Because the diurnal dynamic of the microbiome can affect that of the hepatic transcriptome (Leone et al., 2015; Manella et al., 2021; Weger et al., 2019), we hypothesized that the ileal transcriptome would also be affected by these luminal oscillations. Moreover, because the FA condition led to a decrease in the percentage of ASVs that had diurnal fluctuations, and the FT condition dramatically increased this number, we also hypothesized that FT could prevent the loss of daily cyclical dynamics of the ileal transcriptome. Ileal transcriptome analysis by RNA sequencing (RNA-seq) followed by rhythmic analysis using MetaCycle revealed that the overall number of cycling transcripts was dramatically decreased in FA compared with NA, and partially maintained in FT (Figures 4A and 4B). We observed that 1,862 protein-coding genes had circadian cycling across all three conditions (Figure 4B). However, closer analysis of these transcripts showed that HFD still disrupted their rhythms in FA mice by inducing a phase shift toward the light period (Figures 4C and 4D). Notably, TRF partially maintained the phase of these transcripts in the FT mice (Figures 4C and 4D). This suggests that rhythmic luminal dynamics is related to circadian maintenance of TRF on the host peripheral clock.

Since the FA condition disrupts cycling of a number of genes, we interrogated which genes had their cycling maintained by TRF. An enrichment analysis based on shared cyclical genes between NA and FT, but not FA, indicated that the significant Gene Ontology (GO) (Ashburner et al., 2000; Gene Ontology, 2021) terms maintained by TRF comprise a wide range of terms, including those related to phospholipid metabolism, autophagy, and circadian rhythms (Figure 4E). Conversely, a number of genes gain circadian oscillations in FA only, for which enriched pathways are shown in Figure S4. The fact that TRF maintains diurnal fluctuation of the microbiome and protects mice from the detrimental metabolic effects of HFD suggests that circadian and metabolic genes were important for the effects of TRF on the host intestinal clock. In fact, cyclical dynamics of major circadian genes were disrupted by HFD (Figure 4F). HFD *ad libitum* induced loss of cycling in *Rev-erb*, *Per3*, and *Clock*, all of which were maintained in TRF (Figure 4F). A change in phase was also observed for *Bmal1* and *Cry1* in FA but maintained in FT (Figure 4F) according to phase estimates from MetaCycle (Wu et al., 2016). Contrary to what was observed for these circadian genes, a loss of rhythmicity is observed in *Cry2* under the FT condition. In addition, *Ppara* and *Nfil3*, circadian regulatory genes that are modulated by the gut microbiota (Mukherji et al., 2013; Wang et al., 2017), were also dampened in the FA condition through phase shift and disrupted cycling, respectively, but were maintained in the FT condition (Figure 4F). Hence, TRF prevention of obesity and dysmetabolism occurs in the setting of maintained ileal circadian cycling, maintenance of the phase of cycling in the clock genes, and maintained luminal diurnal dynamics. Since inconsistencies in previous studies of the intestinal transcriptome could be explained by studies not accounting for circadian timing of their target genes, we have created an interactive tool to allow investigators to determine the cycling and phase of their target genes and additional RNA-seq data at https://zarrinparlab.github.io/ti_cycling_paper/ti_expression.html (Figure S3).

### TRF reprograms host transcriptome in the ileum

**Figure 5.**
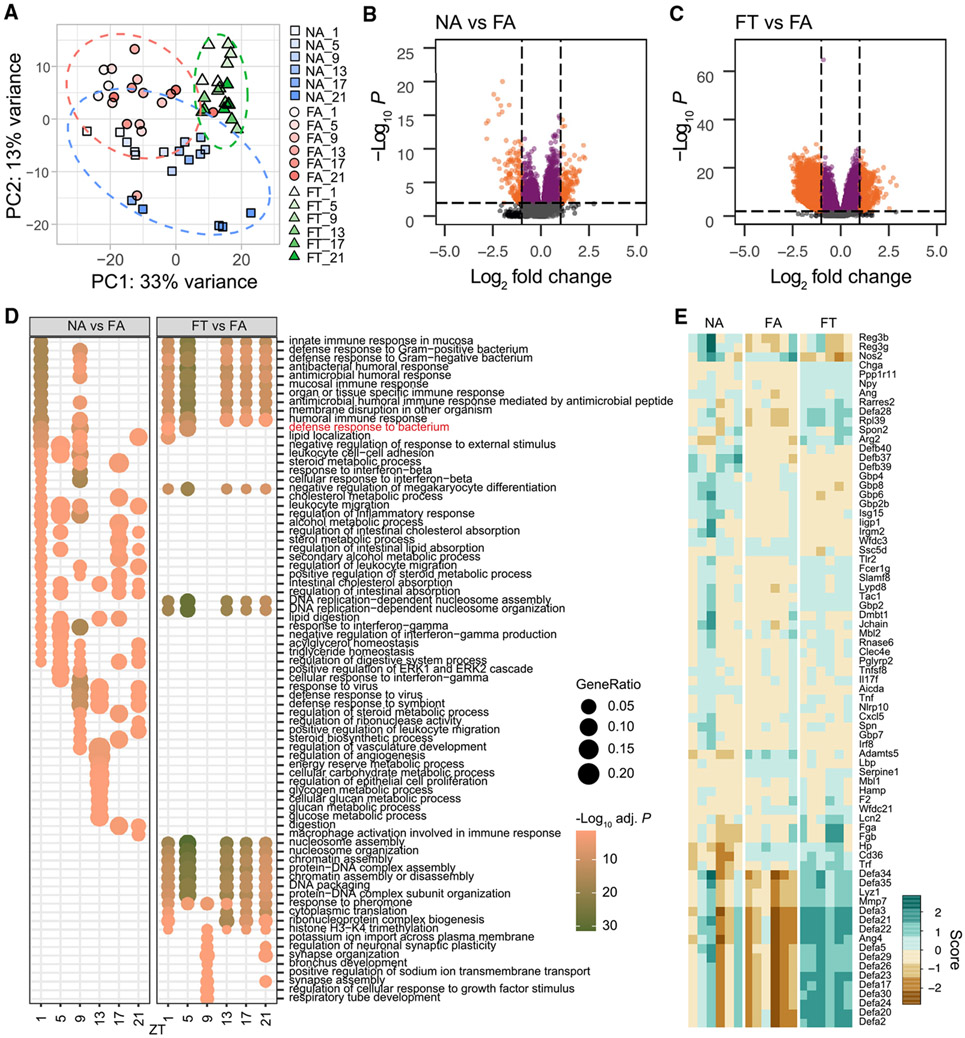
Differential gene expression analysis (A) PCA of transcriptome shows clustering of transcripts by condition (diet + feeding patterns) with variations with time of the day. Ellipses show 95% confidence level for a group of points. (B and C) Volcano plots show log_2_ fold change (LFC) of gene expression between conditions and −log_10_ of p values. Dashed lines represent an absolute LFC cutoff of 1.0 (vertical lines), or −log_10_(p value) of 2 (horizontal line). Data point colors are based on the following criteria: orange represents significant p value and above LFC cutoff; purple represents significant p value; dark gray represents above absolute LFC cutoff; light gray represents not significant. Number of upregulated and downregulated transcripts based on cutoffs are 160 and 203 respectively for NA versus FA; and 600 and 5984 respectively for FT versus FA. (D) Over-represented GO annotations obtained from differentially expressed (DE) genes between conditions, by time point. The GO annotation “Defense response to bacterium” is highlighted in red and further shown in (E). (E) Heatmap of normalized expression of DE genes based on the GO term defense response to bacteria. Scores are based on vst-transformed values (Love et al., 2014). Three mice per time point were used for each condition, for a total of NA (n = 18), FA (n = 18), FT (n = 18).

Having observed that DIO (i.e., the FA condition) dampens diurnal fluctuation of the luminal microbiota and the ileal transcriptome, and that TRF (i.e., the FT condition) maintains microbiome and host transcriptome circadianness (Figures 1 and 4), we investigated the altered products and potential pathways through which the ileum can contribute to the TRF metabolic outcomes. Visual inspection of principal component analysis (PCA) of transcriptome data suggests that mice ileum samples are distinguished under different diet and feeding conditions (Figure 5A), which could indicate that metabolic phenotype alone does not dictate transcriptional activity in the ileum. To determine which transcripts were involved in modulating the similar lean, insulin sensitive metabolic phenotype in FT and NA conditions, we determined differentially expressed (DE) genes between NA versus FA and FT versus FA mice. Overall, TRF had a greater effect on gene expression with an increased number of DE genes between FT and FA compared with NA and FA in the ileum (Figures 5B and 5C), a phenomenon that is observed across different time points (Figure S5). Hence, even though diet was the same between FA and FT, and the microbiome was less different, the feeding pattern change had a bigger impact on the ileal transcriptome than the lean phenotype itself.

We next determined which pathways were involved in the phenotypic differences observed in our study by performing an over-representation analysis of DE genes in FT versus FA and NA versus FA, since these can be potential targets for the treatment of obesity and diabetes and could explain how TRF maintains metabolic homeostasis in the face of a nutritional challenge. HFD *ad libitum* disrupts the transcriptome with dynamic circadian changes in DE genes and over-represented GO terms (Figure 5D, NA versus FA). This is mostly driven by genes involved in digestion, metabolism, and defense response. Surprisingly, in HFD, over-represented terms between FT and FA mostly displayed constitutive patterns throughout the light and dark periods. This suggests that restricting HFD access by TRF induces transcriptional changes that are inherently different from those observed by a change in diet alone (Figure 5D, FT versus FA). The majority of over-represented terms between FT and FA are related to defense response and chromosome organization. Overall, TRF acts on immune-related pathways and reprogramming specific nucleosome and chromatin ones (Figure S6). Genes involved in immune response to bacterium (Figures 5D and 5E) have decreased expression levels in FA, particularly α-defensins, whose major components are secreted by Paneth cells in response to bacteria stimuli (Ayabe et al., 2000; Ouellette, 2010). FT maintained high expression levels of α-defensins, demonstrating how feeding pattern can have a profound effect on host ileal gene expression.

**Figure 6.**
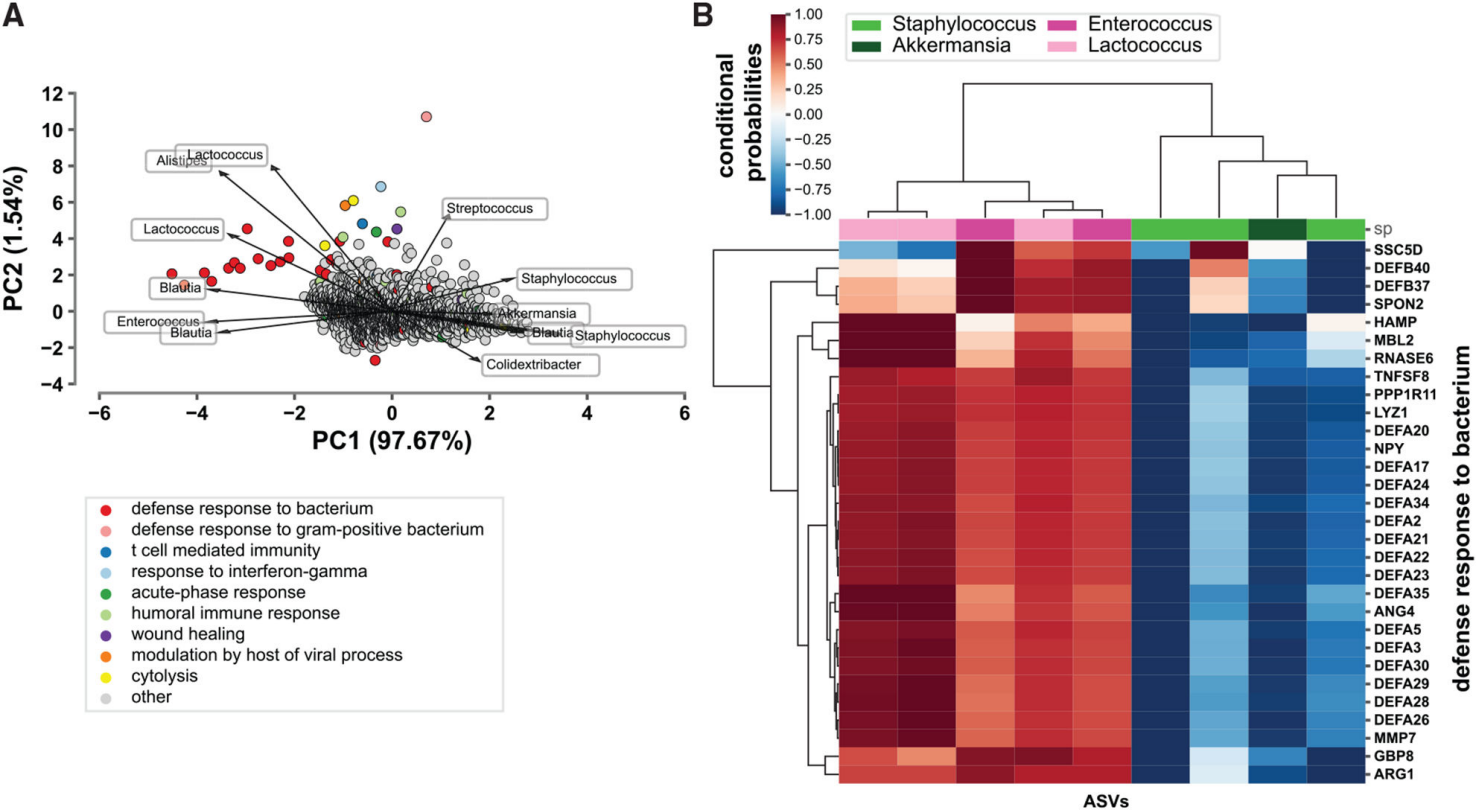
Relationship between host transcriptome and microbiome (A) Biplot representing co-occurrence probabilities between ileal host transcripts and gut microbes. Principal components (PCs) PC1 and PC2 based on mmvec conditional probabilities are represented. Points and arrows represent specific transcripts and microbes, respectively. Direction of arrows represent co-occurrence patterns between microbe and transcript. Color of points represent specific GO terms that transcripts belong to. (B) Heatmap showing snapshot of conditional probabilities between ASVs and host transcripts part of the defense response to bacterium GO term. The families of identified ASVs are denoted by the legend. Three mice per time point were used for each condition, for a total of NA (n = 18), FA (n = 18), FT (n = 18).

To investigate host-microbe relationships further, we performed a co-occurrence analysis between the host transcriptome and microbiome within the ileum using mmvec (Morton et al., 2019b). Major co-occurrence probabilities depict possible relationships between the host transcriptome and microbiome (Figures 6A and 6B). Transcripts belonging to the GO term defense response to bacterium display a strong relationship with specific microbes (Figure 6A). There is a high conditional probability that these transcripts co-occur with *Lactococcus* and *Enterococcus* species, with the opposite trend being observed for *Staphylococcus* and *Akkermansia* (Figure 6B). The biological significance of these findings requires additional *in vivo* validation. Collectively, transcriptome analysis of the ileum of NA, FA, and FT mice suggests that diet and feeding pattern modulate distinct effects on host transcriptional networks. Most enriched GO terms between NA and FA conditions act in a circadian manner through regulation of digestive, lipid, metabolic, and immune pathways. Enriched terms between FT and FA, on the other hand, are observed as constitutive, suggesting that TRF acts as a different entity that could remedy the dysmetabolism induced by HFD through the modulation of a specific set of genes different from those disrupted by HFD alone in FA mice.

### TRF alters GLP-1 and bile acid signaling

**Figure 7.**
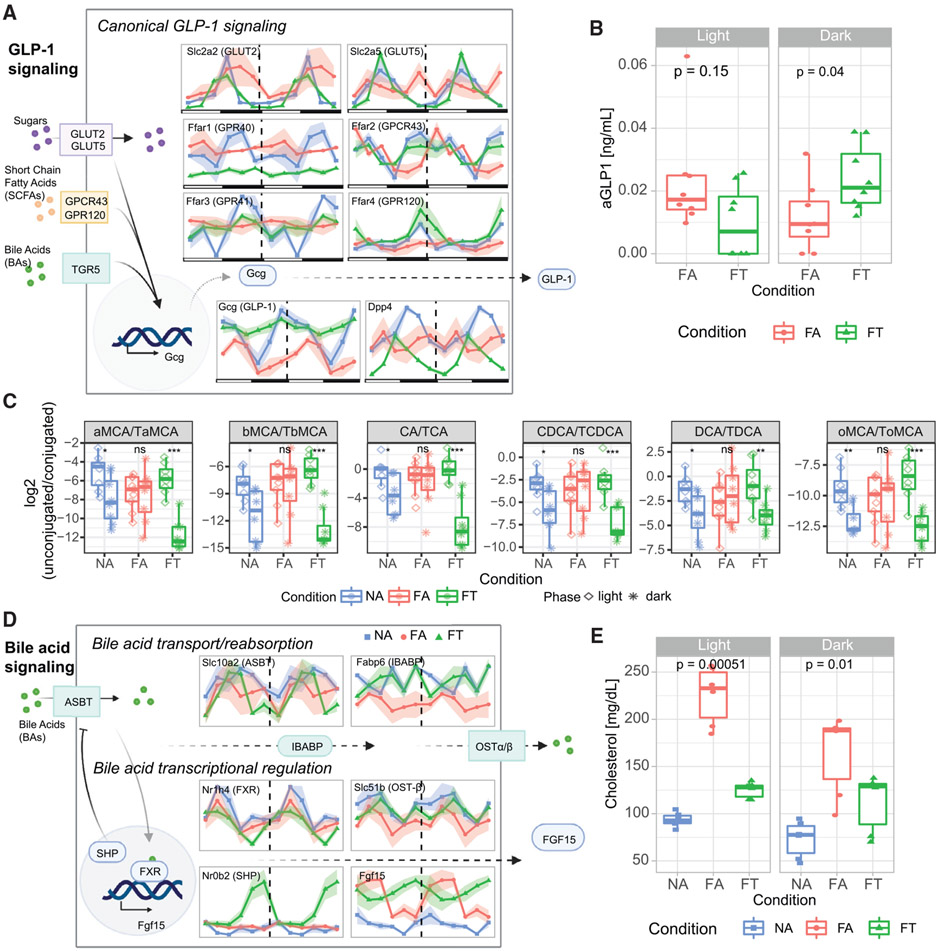
Disruption of metabolic signaling pathways of the ileum (A) Schematic representation of expression levels of GLP-1 signaling pathway genes. (B) Plasma active GLP-1 (aGLP-1) levels in mice fed HFD under FA or FT conditions. (C) Ratios of unconjugated to conjugated bile acids in light and dark phases for each condition. (D) Schematic representation of expression levels of bile acid signaling pathway genes. (E) Serum cholesterol levels under different feeding conditions. Transcript levels are expressed as TPM. Please see Figure S7 for figures with y axis measures. NA = blue, FA = red, FT = green. Three mice per time point were used for each condition, for a total of NA (n = 18), FA (n = 18), FT (n = 18) in (A) and (B). Shading areas show standard error of mean (SEM).

Since TRF improves insulin sensitivity and adiposity in mice consuming an HFD (Figure S1A), we investigated how this feeding pattern affected intestinal metabolic signaling pathways in the context of preserved diurnal oscillation of the gut microbiome and ileal clock (Figures 4D-4F). Here we specifically focus on pathways related to GLP-1, which is a major glucoregulatory ileal hormone, and fibroblast growth factor 15 (FGF-15), which is released with FXR activation (Figure 7) (Drucker, 2018; Gadaleta and Moschetta, 2019; Zarrinpar et al., 2014; Zarrinpar and Loomba, 2012).

The expression level of the proglucagon gene *Gcg* (a precursor of GLP-1) is dampened, and arrhythmic in the FA condition (Figures 7A and S7). However, TRF elevates *Gcg* expression levels and maintains its circadianness in FT mice. These changes in *Gcg* in FT mice are accompanied with decreased expression of *Dpp4*, which encodes the enzyme that degrades GLP-1 and plays a key role in glucose metabolism (Figures 7A and S7). Ileal *Gcg* expression is increased when there are more nutrients (e.g., glucose, free fatty acids/short-chain fatty acids [SCFAs]) in the distal gut. *Ad libitum* consumption of HFD led to phase shifts of the gene expression of the transporters of these nutrients, such as GLUT5 (*Slc2a5*) and GLUT2 (*Slc2a2*) for glucose and GPR40 (*Ffar1*), GPCR43 (*Ffar2*) and GPR120 (*Ffar4*) for FFAs/SCFAs. Moreover, it also led to dampening of *Ffar4* expression and a decreased *Ffar3* amplitude. The expression of most of these transporters in FT mice is indistinguishable from NA mice, demonstrating how the feeding pattern is a major modulator of normal expression of these genes. Moreover, increased expression of these genes may explain why *Gcg* expression is persistently elevated in FT compared with FA mice.

To determine if these gene expression changes affected actual incretin levels, we measured active GLP-1 (aGLP-1) serum levels in dark and light phases of FA and FT mice. Importantly, as suggested by the transcriptomics results, the aGLP-1 levels were 2-fold higher in the dark phase in FT mice (during feeding; p = 0.04) compared with FA mice. However, during the light phase, when FT mice are not consuming any food, aGLP-1 was lower and not significantly different from that measured in FA mice (p = 0.15; Figure 7B). Another glucoregulatory hormone released at the ileum is peptide tyrosine-tyrosine (PYY), which signals satiety and decreases food intake. *Pyy* expression was similar in FA mice compared with what was observed in NA mice. Expression in FT mice, however, was predominantly increased (Figure S7A), although it should be noted that FT mice do not have altered food intake (Chaix et al., 2014; Hatori et al., 2012).

Along the bile acid-FXR-FGF15 signaling pathway, which regulates enterohepatic bile acid response, the FA condition dramatically affects bile acid signaling (Figures 7C and S7B). First, compared with NA mice, the FA mice had altered unconjugated to conjugated bile acid ratios between light and dark phases (Figure 7C). However, every single pair of unconjugated/conjugated bile acid becomes similar to NA in the FT condition, which is primarily driven by the variation of unconjugated bile acids (Figure S7B). Second, a number of transcripts in the bile acid-FXR-FGF15 pathway are disrupted in gene expression levels and circadian rhythmicity (Figures 7D and S7A). Transcriptional regulation of different components is partially regulated in enterocytes by FXR (encoded by *Nr1h4* gene). Surprisingly, across NA, FA, and FT, gene expression levels of *Nr1h4* are the same (Figures 7D and S7), despite previously observed bile acid homeostatic disruptions in HFD (Zarrinpar et al., 2014). This is consistent with what we have observed in the liver, despite the differential regulation of many downstream hepatic targets of FXR (Hatori et al., 2012). However, unlike in the liver, we noted a dampening of *Nr1h4* circadian rhythmicity in FA, which is maintained in FT mice (Figures 7D and S7). *Fgf15*, a critical component of the bile acid signaling pathway, displays cyclical patterns across all three conditions, but increased amplitudes under HFD conditions (Figures 7D and S7). One of its regulators, SHP, is encoded by *Nr0b2*, a downstream target of FXR, which is dramatically overexpressed in FT animals compared with both NA and FA. Specifically, robust cycling and higher amplitude are observed for *Fgf15* in FA mice. This is contrary to what is observed in NA, for which expression levels are lower than both FA and FT. In addition, circadian gene expression of bile acid transporters *Fabp6* (IBABP), *Slc51a* (OST-α), and *Slc51b* (OST-β) were altered by HFD, while *Slc10a2* (ISBT) remained unaltered (Figures 7D and S7). TRF led to maintenance of gene expression levels for *Fabp6* and circadian rhythmicity for *Slc51a* and *Slc51b*. Ultimately, ileal bile acid signaling controls *de novo* bile acid synthesis from cholesterol by CYP7A1 (Chiang, 2009). We had previously shown hepatic expression of *Cyp7a1* to be suppressed in FA mice that also have elevated serum cholesterol levels, whereas TRF preserved normal *Cyp7a1* expression and cholesterol homeostasis (Figure 7E) (Hatori et al., 2012; Zarrinpar et al., 2014). Additional gene expression levels of transcripts related to the metabolic phenotypes studied here are depicted in Figure S7 and can be searched with our online tool (see Figure S3).

Overall, our results suggest that GLP-1 and bile acid signaling are compromised under DIO but maintained by TRF. *Gcg* and *Dpp4* lose circadian rhythmicity and have their expression levels decreased when mice are given *ad libitum* access to HFD. TRF, however, maintains circadian dynamics of both and led to changes in gene expression for additional transporters involved in GLP-1 signaling. Furthermore, bile acid pool, transport, reabsorption, and signaling are compromised under DIO but maintained by TRF. Bile acid signaling in hepatocytes is likely disrupted by DIO, with increased oscillations in mice fed an *ad libitum* diet compared with normal or TRF conditions.

## DISCUSSION

The gut microbiome is intimately related to circadian rhythms, and dysregulation of major circadian genes as well as time of feeding are disruptors of the gut microbiome in animal models (Bishehsari et al., 2020; Choi et al., 2021; Frazier and Chang, 2019). What is less understood is how the gut microbiome and oscillations in its daily rhythms contribute to host phenotype and physiology, particularly in the ileum, which is a major region responsible for regulating host metabolic signaling and physiology. We address this gap in knowledge with an omics approach in a mouse model of DIO and a model of TRF, which prevents HFD-induced weight gain and dysmetabolism, in spite of both models consuming the same amount of calories (Chaix et al., 2014; Hatori et al., 2012). Our results reveal disrupted rhythms on microbiome components and transcriptome pathways, and identify potential functional ways through which some of the metabolic benefits of TRF could be mediated. DIO disrupts the total number of cyclical ASVs and transcripts to a greater extent than TRF, suggesting that TRF alone alleviates some of the effects of DIO, potentially by modulating the intestinal clock. This opens a paradigm where entrainment of the peripheral clock could be modulated by the ileum.

Interestingly, the ileal microbiome is very dynamic in TRF, with an increased percentage of ASVs cycling and more reads belonging to these ASVs (Figure 1F). Nevertheless, diet remains a powerful influence in the composition and dynamics of the ileal gut microbiome as demonstrated by compositional and α-diversity similarities between the two HFD conditions, which is consistent with what we had reported in the cecum (Zarrinpar et al., 2014). Despite different techniques used for the cecal and ileal microbiomes analysis, the overall conclusions of each experiment stand independently and highlight how circadian oscillations vary in the ileum and cecum based on diet and feeding conditions.

TRF could exert its effects by modulating changes of specific bacteria levels involved in metabolic processes. Additional factors, such as food consumption and behavior, are also apparent from our microbiome data. For instance, *Lactococcus*—a contaminant present in irradiated high-fat food (Bisanz et al., 2019) —exhibits a clear pattern based on the feeding schedule of our mice. Also, *Staphylococcus*, which is prevalent on the murine skin (Belheouane et al., 2020), has an increased pattern in FT and NA mice, and could point toward grooming behavior in these animals prior to feeding. Intriguingly, it is unclear if these bacterial “contaminants,” whether they be from food or skin, play a role in eliciting a physiological response. This is relevant because microbiota organisms can act as entraining agents of daily rhythms, as recently reported for segmented filamentous bacteria diurnal oscillations and daily rhythms in innate immunity through the expression of antimicrobial proteins (Brooks et al., 2021). We plan to use recently developed tools to interrogate whether TRF-induced microbiome changes, such as the changes in conjugated and unconjugated bile acids, induce host metabolic change through functional manipulation of the gut microbiome in conventionally raised mice. A study in full conventional mice with engineered native bacteria that express bile salt hydrolase (BSH), an enzyme from the gut microbiome important for bile acid metabolism, shows that BSH affects glucose homeostasis (Russell et al. in press). Thus, additional studies that allow for functional manipulation of the gut microbiome will allow us to determine contributions and causality of individual bacteria or bacterial functions on the mechanisms of action of TRF.

The results of CDKO mice show that an intact host circadian clock is required for normal ileal microbiome dynamics and is consistent with previous reports of microbiome circadian disruptions when using whole-body knockouts of clock genes *Bmal1* and *Per* (Liang et al., 2015; Thaiss et al., 2016). TRF protects CDKO animals from metabolic disruptions (Chaix et al., 2019a). Moreover, restricted feeding recovers some transcriptional activity that had been lost in these animals (Chaix et al., 2019a; Weger et al., 2021). Together, these studies further advance the theory that the central clock’s main method of regulating peripheral clocks is likely through regulating feeding behavior and, with it, microbiome dynamics, as opposed to an as-yet unidentified neurohumoral mechanism (Frazier and Chang, 2019).

Studies in germ-free and antibiotic-treated mice indicate that the microbiome is necessary for peripheral circadian rhythms in the intestinal and hepatic clock and even in hypothalamic nuclei (Leone et al., 2015; Weger et al., 2019). For example, modulation of the small intestine microbiome through AIMD led to loss of *Per2* rhythmicity (Martchenko et al., 2021). Previous work also showed that DIO mice have dyssynchronous hepatic circadian rhythms in the setting of disrupted cecal microbiome rhythms (Leone et al., 2015). We extend this work by showing that ileal microbiome and transcriptome circadian rhythms are also disrupted in DIO. Moreover, we show that TRF (Chaix et al., 2019b) has a corrective effect on the ileal microbiome rhythms and maintains ileal peripheral rhythms, particularly to the circadian clock genes. However, it is unclear which secondary metabolites or proteins can act as entraining agents to the peripheral clock, although SCFAs and deconjugate bile acids have been suggested by some studies (Bishehsari et al., 2020; Choi et al., 2021; Frazier and Chang, 2019).

Transcriptomic analysis indicates that DIO perturbs the intestinal clock and metabolic pathways of the ileum, which in turn are maintained by TRF. For example, genes encoding SCFAs (Tolhurst et al., 2012) and fructose transporters (Ferraris et al., 2018), whose activations are involved in stimulating GLP-1 secretion, are dampened in DIO (Figure 7A). Increased levels of *Pyy* with TRF could explain why early studies of TRF show caloric restriction or reduced appetite in humans (Gill and Panda, 2015; Sutton et al., 2018; Wilkinson et al., 2020). DIO dampens circadian rhythms not only through loss of cyclical patterns but also through more nuanced changes in circadian gene expression such as amplitude and phase shifts (Figures 4C and 4D). TRF, on the other hand, prevents the dampening or perturbation of these rhythms. TRF mice display constitutive changes in the ileum transcriptome in addition to preventing the DIO dysmetabolic phenotype and liver dyssynchronous rhythms (Hatori et al., 2012). This suggests that TRF acts not only through the maintenance of pathways disrupted under DIO but also through a unique response to circadian changes in feeding pattern. Our results suggest that immuno-regulatory pathways are related to differences in TRF/DIO response. Thus, studies with *Mmp7*−/− mice might help elucidate some of the molecular mechanisms of TRF response such as regulation of alpha-defensins.

To better understand the relationship between the microbiome and host transcriptome, we performed a trans-omic co-occurrence analysis where we found strong conditional probabilities of specific bacterial families and genes co-occurring (Figure 6). This strongly suggests that relationships between ileal microbes and host transcripts exist, although this relationship might not be specific to the ileum. For instance, modulation of SCFAs through oscillation of the cecal microbiota affects colon transcriptome regulation (Arora et al., 2019). Thus, the biological implication of our findings requires further investigation. Multi-omic analyses using a variety of dietary, gut regional, host phenotype, feeding pattern, and collection time points will help eliminate spurious findings and build more accurate models that can be better interrogated over time.

Our findings further highlight potential implications of regional differences in circadian dynamics and the need for better circadian phenotype, transcriptome, and microbiome characterization. It is conceivable that two investigators performing the same exact study in similar settings may come to different conclusions solely based on the timing of their sample collection. This is particularly important with microbiome analysis (Allaband et al., 2021). Moreover, it is possible that specific interventions or drugs may only be effective at specific times due to availability of xenobiotics or the amplitude of key metabolizing genes, which can alter results significantly between studies. This, in turn, prompted us to provide a Web resource of circadian microbiome and transcriptome data that can be easily queried. This resource could aid in explaining discrepancies obtained across similar experiments and enable investigators to make better-informed decisions about experiments, since oscillations within daily patterns vary greatly and can influence interpretation of results.

### Limitations of the study

Our microbiome and transcriptome ileal results demonstrate that TRF acts through maintenance of diurnal fluctuation and the restoration of host phenotype. Nonetheless, duodenal and jejunal tissue, as well as nutrients absorbed in these regions, likely play an important role in metabolism despite their lower microbial and secondary metabolites content. Thus, additional studies targeting multiple GI tissues are needed to disentangle the contributions of TRF on metabolism and circadian rhythms. Furthermore, different sequencing techniques were used for ileal and cecal microbiome experiments, which made comparisons of these regions difficult to perform. Moreover, the CDKO mice are of different maternal line than control mice, which could affect the composition of the gut microbiome, again making comparisons between conditions difficult to perform. As demonstrated in our transcriptomic analysis, TRF affects many physiological processes. Although we have focused primarily on bile acid and incretin signaling, it is possible that other processes, such as inflammation, gut barrier function, and autophagy, which are also affected by TRF, could play as important a role, which we hope other investigators will explore. Although we have used the most state-of-the-art bioinformatic tools available to study the relationship between the gut microbiome and host transcription as part of our multi-omic analysis, these findings are still associative and require additional investigation. We plan to use functional manipulation of the gut microbiome through engineered native bacteria (Russell et al. in press) to allow us to mechanistically investigate the relationship between specific bacterial functions and host physiological processes.

## STAR★METHODS

### RESOURCE AVAILABILITY

#### Lead contact

Requests for further information should be directed to and will be fulfilled by the lead contact, Amir Zarrinpar (azarrinpar@ucsd.edu).

#### Materials availability

This study did not generate new unique reagents. Samples might not be available due to small amounts obtained from mouse experiments.

#### Data and code availability

- Raw sequencing data derived from RNA-seq and 16S rRNA amplicon sequencing has been deposited at the European Nucleotide Archive (ENA). Accession numbers are listed in the key resources table. Processed 16S and RNA-seq data, targeted metabolomics data, and physiological data have been deposited to Mendeley. The DOI is listed in the key resources table. Data is publicly available as of date of publication. This paper analyzes existing data. The source for the datasets is listed in the key resources table.
- All original code has been deposited to Mendeley and is publicly available as of the date of publication. DOI is listed in the key resources table.
- Any additional information required to reanalyze the data reported in this paper is available from the lead contact upon request.

### EXPERIMENTAL MODEL AND SUBJECT DETAILS

#### Animals and tissue collection

All animal work was approved by the Salk Research Institute IACUC. All experiments conform to current regulatory standards. Mice were the same as described in Zarrinpar et al. (2014), with a total of 54 8-week-old, wild-type male C57BL/6 mice (Jackson Laboratories, Bar Harbor, ME) subject to different diet and food access patterns for 8 weeks as previously described (Zarrinpar et al., 2014). Mice were split into three groups: mice fed a NCD with *ad libitum* food access (n = 18, NA condition), a HFD with *ad libitum* food access (n = 18, FA condition), or a HFD with time-restricted food access (n = 18, FT condition). Time-restricted food access refers to restricting food access to a period of 8 hours during the dark (ZT 13–21). Normal chow (LabDiet 5001): 3.36 kcal/gm; High-fat (TestDiet 58Y1): 5.16 kcal/gm (Zarrinpar et al., 2014). Whole body clock mutant *Cry1*;*Cry2* double KO (CDKO) were obtained from the Sancar lab and were backcrossed to C57/B6 background >5 times. The genotype of the animals was confirmed by PCR (Chaix et al., 2019a; Vitaterna et al., 1999). CDKO mice were kept in similar light tight boxes with *ad libitum* food access (n = 18, CDKO-NA). For every 4h time point, three animals from each condition from separate cages were euthanized and whole ileum (content and mucosa) samples were collected during a 24h period for each of the 6 timepoints on the Zeitgeber time scale (ZT1, ZT4, ZT9, ZT13, ZT17, ZT21). Samples from ileum, cecum and blood were collected and stored at −80°C until further processing. Tissue samples were powdered through mechanical homogenization (mortar and pestle) in liquid nitrogen. For GLP-1 experiments, 12 weeks old male C57BL/6 mice (Jackson Laboratories) were fed 60% HFD (Research Diet D12492) either *ad libitum* or TRF (9 h of access to food during ZT13 – ZT22) for 9 weeks. For each group, blood was collected on BP800 tubes (BD Biosciences #366420) from 2 mice per time point every 3 hours.

### METHOD DETAILS

#### Tissue DNA extraction and 16S amplicon sequencing

DNA was extracted from powdered tissue samples with the QIAmp DNA Stool Mini Kit (Qiagen). Sample quality and quantity was assessed prior to preparation for 16S rRNA gene amplicon sequencing. The polymerase chain reaction (PCR) was performed as follows for amplification of the 16 S rRNA gene from ileal samples: initial denaturation at 95°C for 5 min, followed by a 31-cycles of 98°C for 20 s; 55°C for 20s; 72°C for 20 s, and a final extension at 72°C for 1 min. All oligonucleotides utilized were obtained from Integrated DNA Technologies (IDT). We performed library preparation and paired-end amplicon 16S sequencing of the ileum samples based on the V3-V4 region using an available protocol on the MiSeq Illumina platform.

#### Tissue RNA extraction and preparation for RNA-seq

Powdered tissue samples were homogenized in TRIzol Reagent (Life Technologies #15596026). RNA was isolated with PureLink RNA mini kit (Life Technologies #12183025) according to the manufacturer’s instructions. Sample quality control and library preparation were performed by the IGM core at UC San Diego. RNA ScreenTape® was used to assess RNA quality and quantity. Sequencing library was obtained based on mRNA. Sequencing was performed on the NovaSeq Illumina platform using 150PE reads.

#### Metabolic measurements

Serum from fasting animals (n = 6) at 8 weeks after diet change/intervention was used for cholesterol using Thermo Scientific Infinity Reagents according to the manufacturer’s instructions. Meso Scale Diagnostics K150JWC kit was used to measure active GLP-1 (aGLP-1) from non-fasted serum.

#### Tissue bile acids

Analysis of bile acids was carried out through liquid chromatography followed by mass spectrometry (LC/MS). Bile acids were extracted from samples according to (Wegner et al., 2017). Briefly, tissue samples were homogenized and extracted in 75% methanol (25 mg of tissue/200 μL) containing heavy internal standards. Supernatants were transferred to glass vials upon 10 minutes of vortexing followed by 10 min of centrifugation (16,000 × g) at 4°C. Samples were injected and analyzed on a Dionex Ultimate 3000 LC system (Thermo) coupled to a TSQ Quantiva mass spectrometer (Thermo) fitted with a Kinetex C18 reversed phase column (2.6 μm, 150 × 2.1 mmi.d., Phenomenex). The LC solvents used consisted of two solutions: solution A - 0.1% formic acid and 20 mM ammonium acetate in water; solution B - acetonitrile:methanol 3:1 (v/v) containing 0.1% formic acid and 20 mM ammonium acetate. A reversed phase gradient at a flow rate of 0.2 mL/min was utilized, with a gradient consisting of 25–29% B in 1 min, 29–33% B in 14 min, 33–70% B in 15 min, up to 100% B in 1 min, 100% B for 9 min and equilibrated to 25% B for 10 min, for a total run time of 50 min. The injection volume for all samples was 10 μL, the column oven temperature was set to 50 C and the autosampler kept at 4 C. MS analyses were performed using electrospray ionization in positive and negative ion modes, with spay voltages of 3.5 and −3 kV, respectively, ion transfer tube temperature of 325 C, and vaporizer temperature of 275 C. Multiple reaction monitoring (MRM) was performed by using mass transitions between specific parent ions into corresponding fragment ions for each analyte. Targets were quantified using isotopically labeled internal standards in Skyline (MacLean et al., 2010).

### QUANTIFICATION AND STATISTICAL ANALYSIS

#### Microbiome data processing and analysis

Available raw cecal data obtained from V1-V3 16 S rRNA amplicon sequencing on the 454 platform (Zarrinpar et al., 2014) was reanalyzed from our previous publication for comparison. Raw ileum sequencing data was obtained from V3-V4 amplicon sequencing using the Illumina 16S metagenomic sequencing protocol. Reads were pre-filtered by mapping to the mouse genome (GRCm38.p5 release) with BWA (Li and Durbin, 2009) and removing reads (for 454 data) or read-pairs (for Illumina data) with a match. Filtered reads were processed using Qiime2, version 2020.11 (Bolyen et al., 2019). To dereplicate and create ASV tables and representatives, we used the dada2 denoise-pyro plugin for the 454(cecal) samples, and the deblur 16S plugin on the forward read of the Illumina (TI) samples, trimmed to 150bp (sequence overlap between forward and reverse reads was too low to use merged pairs). After that, cecal and TI samples were processed identically. We removed samples with fewer than 500 features, and features in either fewer than 2 samples, or which were annotated as unclassified, eukaryotic, mitochondrial, or plasmid. Taxonomy assignments were performed with the sklearn feature-classifier plugin against the full-length Silva v132 precomputed model. Trees were built using the align-to-tree-mafft-fastree phylogeny plugin. Diversity metrics were created using the core-metrics-phylogenetic diversity plugin, rarefied to a sampling depth of 1000. We used log-ratios to assess statistically significant differences between conditions since it takes into account reference-frames and is independent of microbial load (Morton et al., 2019b).

#### RNA-seq data processing and analysis

Transcript abundance was quantified using kallisto (Bray et al., 2016) with GENCODE release M21 (GRCm38.p6). Differential expression analysis between groups (condition and ZT) of interest was performed using DESeq2 (Love et al., 2014), with fold changes determined using the normal method for all transcripts whose counts were greater than 10 across all samples. Over-representation analysis of GO terms was performed with clusterProfiler (Yu et al., 2012).

#### Microbiome and transcriptome diurnal analysis

Microbiome and transcriptome cycling was determined with the JTK_CYCLE algorithm (Hughes et al., 2010) implemented in Metacycle (p-value < 0.05) (Wu et al., 2016) which has been validated for 24-hour data.

#### Integration of host microbiome and transcriptome

Songbird was used to determine differential rankings of microbes between conditions and light vs. dark phases with a formula of "C(condition, Treatment(‘FA’)) + C(condition, Treatment(‘FT’)) + C(light_dark, Treatment(‘dark’))" and an optimized model with 2000 epochs, resulting in a pseudo Q-squared (1 - average absolute model error/average absolute baseline error) of 0.25 (Morton et al., 2019b). Songbird differentials were visualized at the genus level as previously described (Allaband et al., 2021). To determine the conditional probability that each transcript co-occurs with specific microbes, co-occurrence analysis between microbiome and transcriptome data was carried out using the integrative omics approach mmvec (Morton et al., 2019a) as previously described (Allaband et al., 2021).

#### Statistical analysis of data

Number of samples used per time point and condition are described under “Animals and tissue collection” section and in the caption of each associated figure along with statistical method used for analysis. All analyses were performed in python version 3.6.12 (Python Software, 2020) or R version 4.1.0 (R Core Team, 2021).

### ADDITIONAL RESOURCES

An interactive tool that allows for exploration of additional microbiome and transcriptome data is available at https://zarrinparlab.github.io/ti_cycling_paper/ti_16S_family_abundance.html and https://zarrinparlab.github.io/ti_cycling_paper/ti_expression.html, respectively.

## Supplementary Material

1

## Figures and Tables

**Table:** KEY RESOURCES TABLE

REAGENT or RESOURCE	SOURCE	IDENTIFIER
Biological samples		
Mouse ileum tissue	This paper	N/A
Mouse serum	This paper	N/A
Mouse serum for aGLP-1 measurements	This paper	N/A
Chemicals, peptides, and recombinant proteins		
TRIzol Reagent	Life Technologies	Cat# 15596026
Critical commercial assays		
QIAmp DNA Stool Mini Kit	Qiagen	Cat# 51504
PureLink RNA mini kit	Life Technologies	Cat# 12183025
BP800 blood collection system	BD Biosciences	Cat# 366420
aGLP-1	Meso Scale Discovery	Cat# K150JWC
Infinity Cholesterol	Thermo Fisher Scientific	Cat# TR13421
Deposited data		
Raw ileal RNA-seq data	This paper	ENA: PRJEB47185
Raw ileal 16S amplicon s equencing data	This paper	ENA: PRJEB47185
Raw cecal 16S amplicon sequencing data	Zarrinpar et al., 2014	Satchidananda Panda Lab
Targeted metabolomics data for bile acids	This paper	Mendeley Data, V1, https://doi.org/10.17632/xyxpvsyvzn.1
Cholesterol data	Zarrinpar et al., 2014	Mendeley Data, V1, https://doi.org/10.17632/xyxpvsyvzn.1
Weight and blood glucose data	Zarrinpar et al., 2014	Mendeley Data, V1, https://doi.org/10.17632/xyxpvsyvzn.1
Active GLP-1 data	This paper	Mendeley Data, V1, https://doi.org/10.17632/xyxpvsyvzn.1
Experimental models: Organisms/strains		
Mouse: C57BL/6J	The Jackson Laboratory	RRID:IMSR_JAX:000664
Mouse: *Cry1;Cry2* double KO (CDKO)	Chaix et al., 2019a; Vitaterna et al., 1999	N/A
Oligonucleotides		
16 S-FWD: TCGTCGGCAGCG TCAGATGTGTATAAGAGACAG CCTACGGGNGGCWGCAG	This paper	N/A
16 S-REV: GTCTCGTGGGCTC GGAGATGTGTATAAGAGACA GGACTACHVGGGTATCTAATCC	This paper	N/A
Software and algorithms		
BWA	Li and Durbin, 2009	N/A
Qiime2 version 2020.11	Bolyen et al., 2019	https://qiime2.org
Kallisto	Bray et al., 2016	N/A
MetaCycle	Wu et al., 2016	https://www.r-project.org/
R version 4.1.0	R Core Team, 2021	https://www.R-project.org/
Python version 3.6.12	Python Software, 2020	http://www.python.org
Code used for data analysis	This paper	Mendeley Data, V1, https://doi.org/10.17632/xyxpvsyvzn.1
Other		
Resource website for microbiome data	This paper	https://zarrinparlab.github.io/ti_cycling_paper/ti_16S_family_abundance.html
Resource website for transcriptome data	This paper	https://zarrinparlab.github.io/ti_cycling_paper/ti_rnaseq_timepoint_expression.html

## Highlights

- The ileal microbiome and transcriptome display diurnal rhythms
- TRF restores ileal circadian rhythms in mice given a high-fat diet
- TRF restores ileal bile acid signaling
- TRF leads to increased levels of *Gcg* and GLP-1 and decreased levels of *Dpp4*

## INCLUSION AND DIVERSITY

One or more of the authors of this paper self-identifies as an underrepresented ethnic minority in science. One or more of the authors of this paper received support from a program designed to increase minority representation in science.
