# Supplementary material for: Diet and feeding pattern modulate diurnal dynamics of the ileal microbiome and transcriptome
Source: Cell Rep. Author manuscript; Available in PMC 2022 Jul 19. (PMC9296000; doi:10.1016/j.celrep.2022.111008)
Supplement: 1 [file NIHMS1821575-supplement-1.pdf]

Cell Reports, Volume *40*

Supplemental information

Diet and feeding pattern modulate diurnal dynamics of the ileal microbiome and transcriptome

Ana Carolina Dantas Machado, Steven D. Brown, Amulya Lingaraju, Vignesh Sivaganesh, Cameron Martino, Amandine Chaix, Peng Zhao, Antonio F.M. Pinto, Max W. Chang, R. Alexander Richter, Alan Saghatelian, Alan R. Saltiel, Rob Knight, Satchidananda Panda, and Amir Zarrinpar

Diet, feeding pattern and host genetics modulate diurnal dynamics of the ileal microbiome and transcriptome

Ana Carolina Dantas Machado[1,2], Steven D. Brown[1,2], Amulya Lingaraju[1,2], Vignesh Sivaganesh[1,2], Cameron Martino[3,4,5], Amandine Chaix[6], Peng Zhao[2,7], Antonio F. M. Pinto[8], Max W. Chang[2], R. Alexander Richter[1,2], Alan Saghatelian[8], Alan R. Saltiel[2,7,9,10], Rob Knight[3,5,11,12], Satchidananda Panda[13], Amir Zarrinpar[1,2,5,10,14*]

******SUPPLEMENTARY MATERIAL******

Content:

Figure S1-7

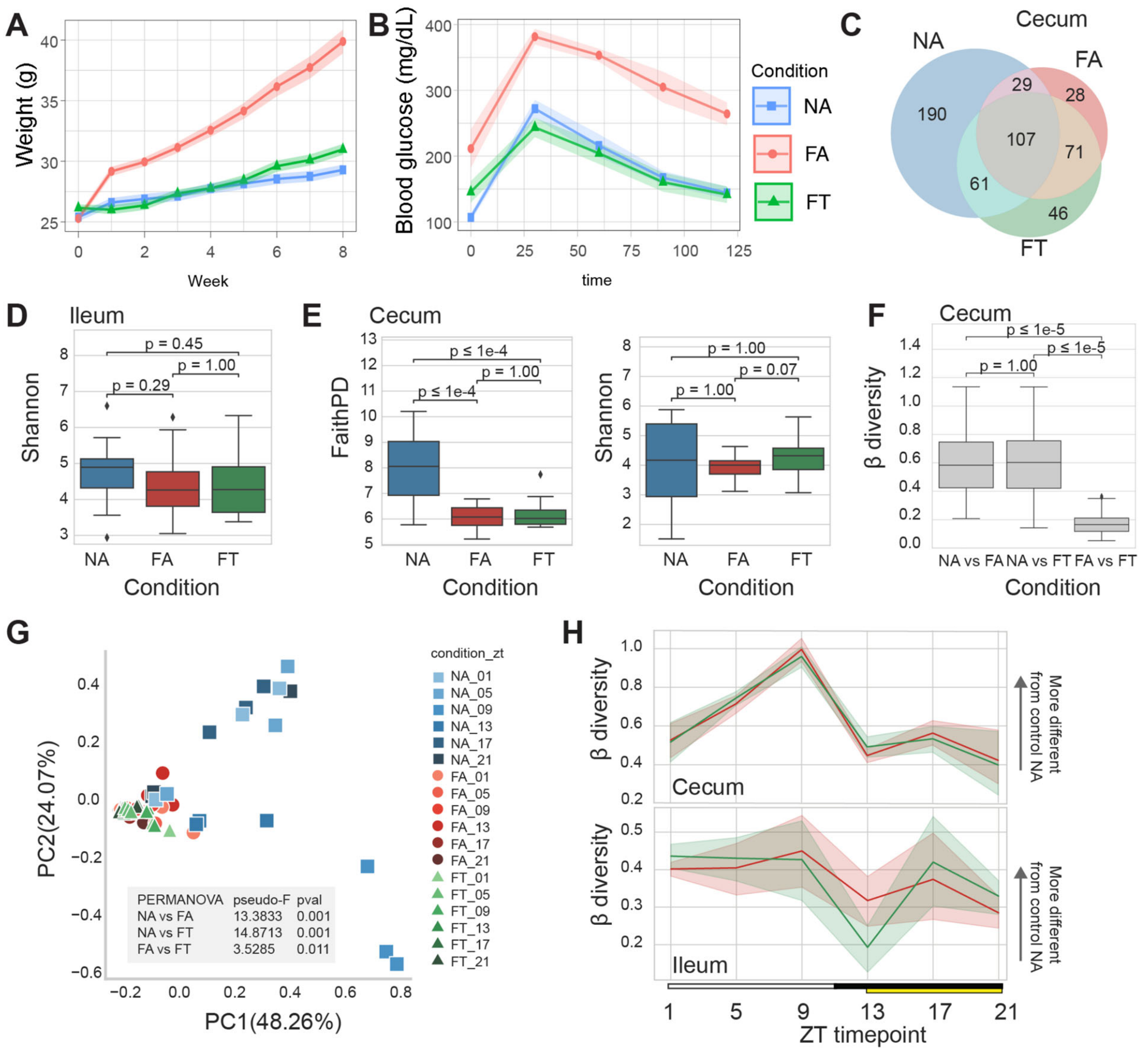

Figure S1. Microbiota diversity of the ileum and cecum, related to Figure 1.

A) Weight and **B)** blood glucose measurements from glucose tolerance test across animals given different diets and feeding conditions (Zarrinpar *et al.*, 2014). Dots/line show average for each condition and shading areas show standard error of mean (SEM).

C) Number of ASVs from cecum samples.

D) Shannon α-diversity of ileum samples.

E) Microbiota diversity measures of cecum samples.

F) Overall β-diversity across conditions in cecum samples.

G) PCoA plot of weighted Unifrac distances. All times are ZT.

H) Time-dependent β-diversity dissimilarity between conditions. Light and dark periods are represented by white and black bars, respectively. TRF food interval is represented by a yellow bar. NA-FA = red; NA-FT = green.

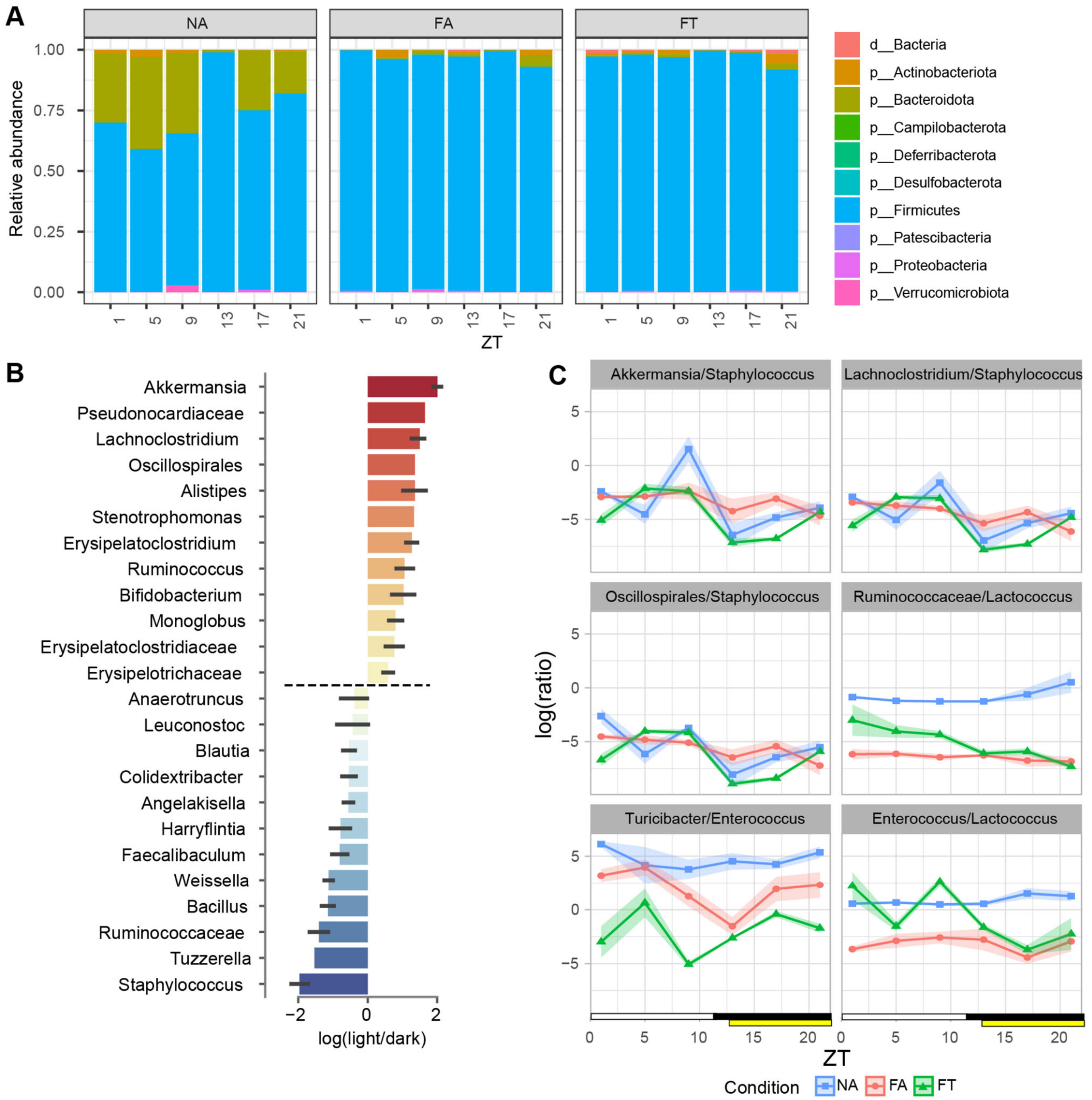

Figure S2. Microbiota composition and cycling dynamics of the ileum, related to Figure 2.

A) Microbiota relative abundances at the phylum level across samples from different feeding conditions and ZT.

B) Differential ranking between light and dark phases as determined by Songbird.

C) Log-ratios of selected comparisons based on highest and lowest rankings from Songbird differentials.

Light and dark periods are represented by white and black horizontal bars, respectively. TRF food interval is represented by a yellow bar.

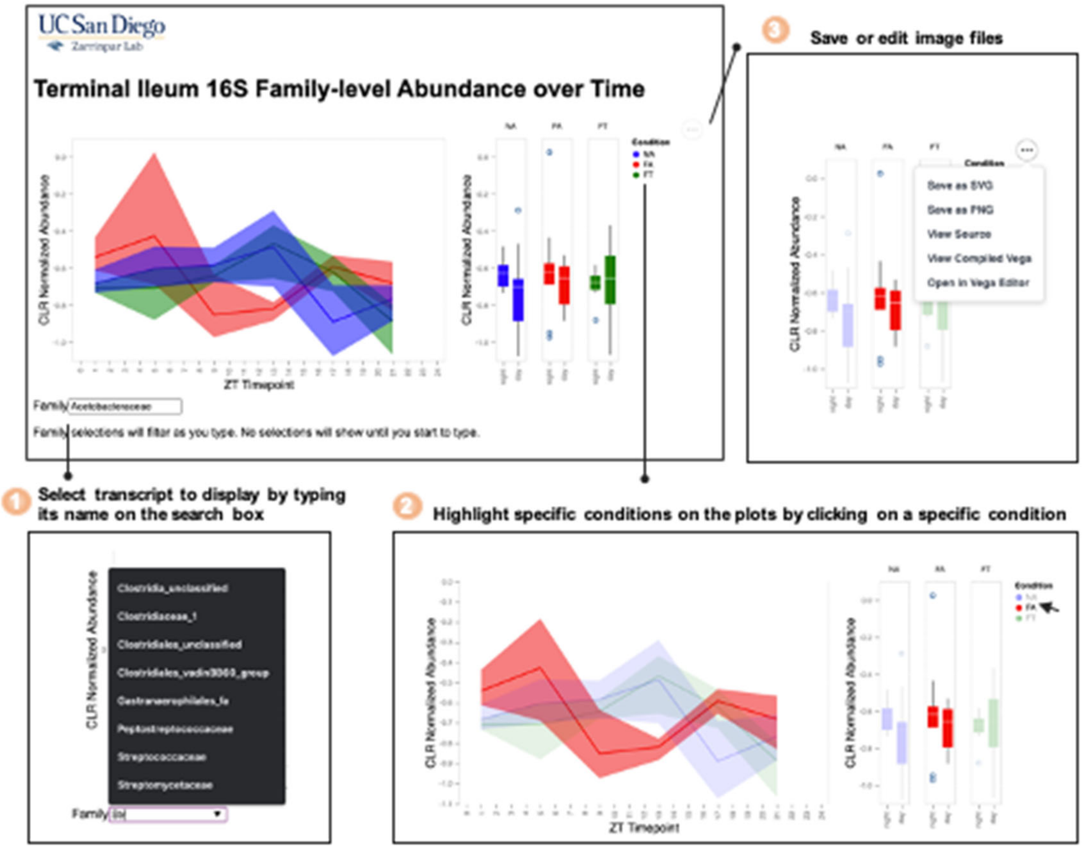

Figure S3. Ileal Bacterial Family Visualization Tool, related to Figure 2. View of web application for visualization of specific microbes in a time-dependent manner. Schematic representation shows how to 1) select specific transcripts of interest; 2) highlight specific conditions and 3) edit and save images.

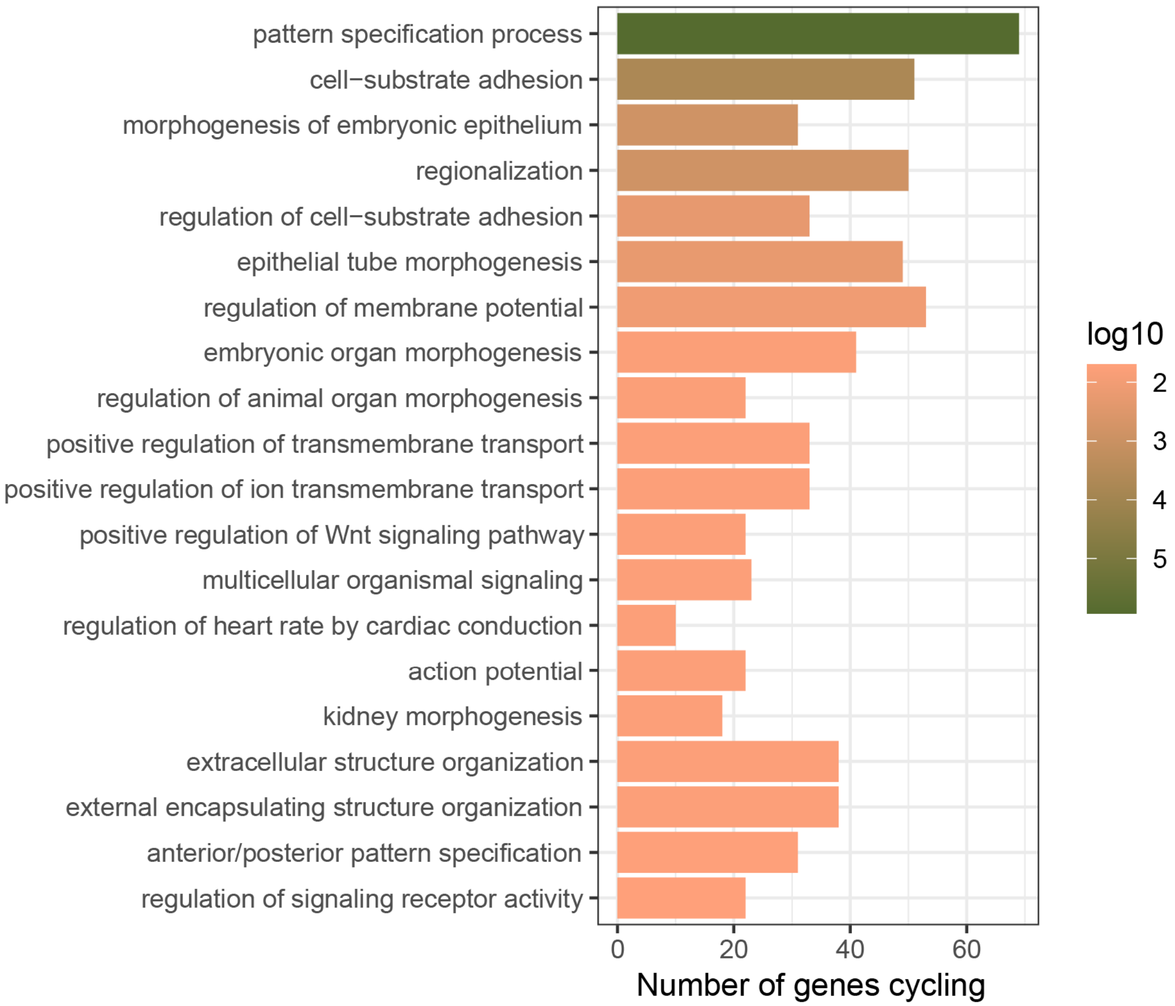

Figure S4. **Over-representation of GO terms related to FA rhythmicity, related to Figure 4**. Enriched GO terms based on genes cycling in FA only.

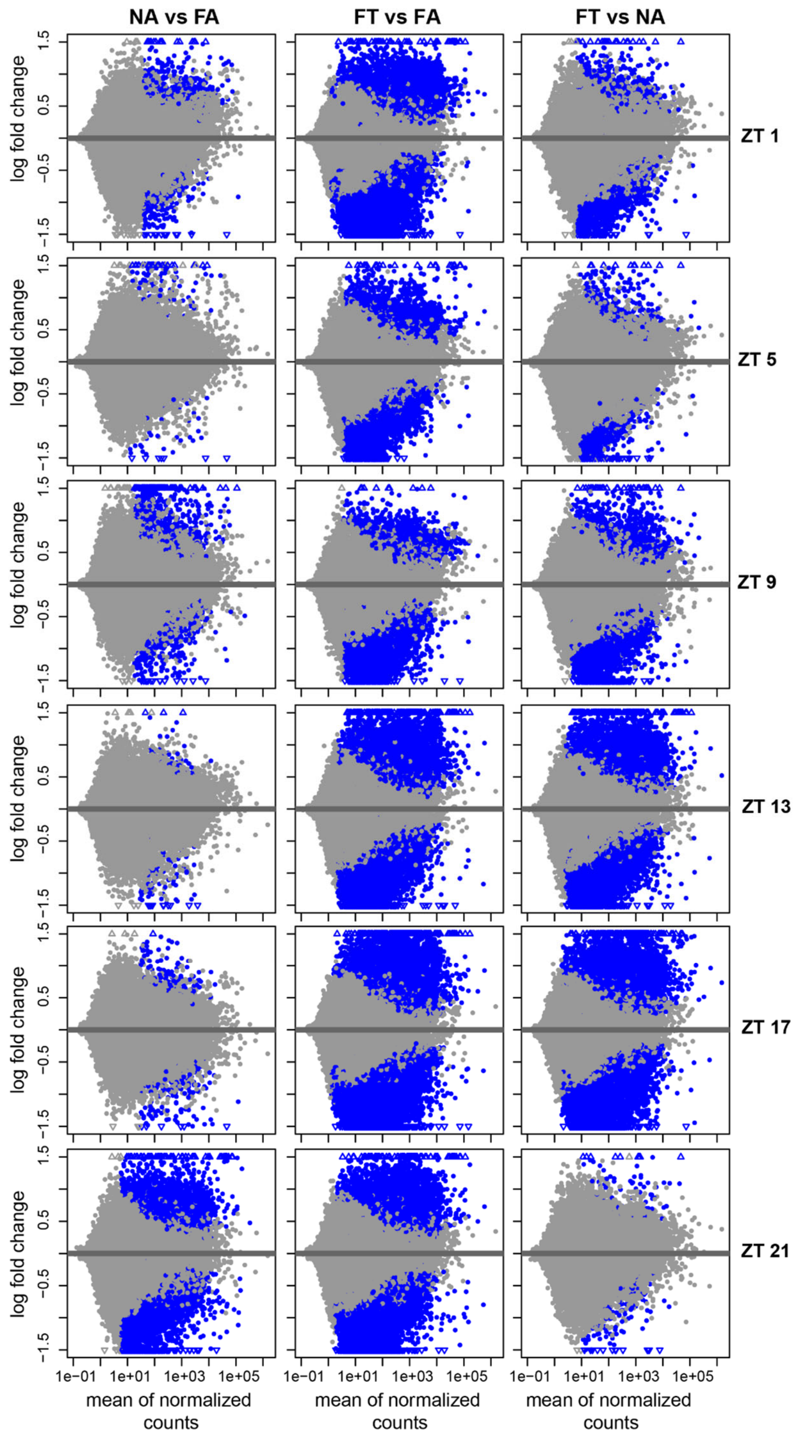

Figure S5. Circadian changes in gene expression over time, related to Figure 5. Log2 fold change of differential expression analysis as calculated by DESeq2. Blue dots represent occurrences with adjusted p-value < 0.1. Open triangles represent points that are out the plotting window. Comparisons are shown for pairs of diet and feeding condition in each timepoint.

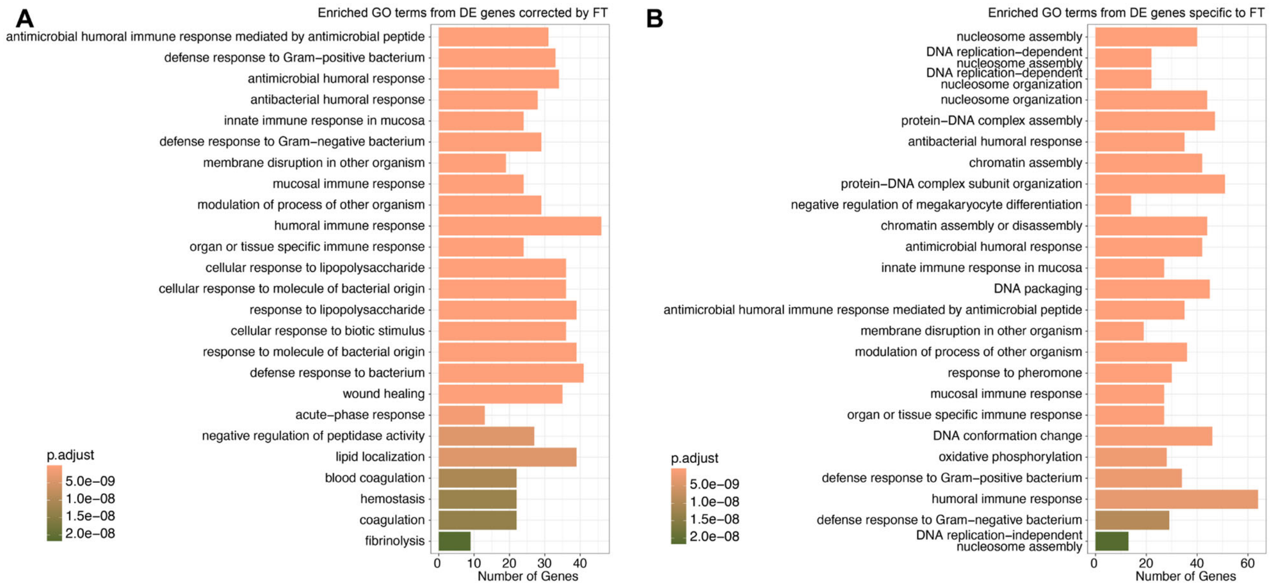

Figure S6. Over-representation of GO terms related to TRF condition, related to Figure 5. Enriched GO terms based on differentially expressed genes (DE genes) which **A)** are corrected by FT in relation to FA or **B)** are specific to FT when compared to both FA and NA.

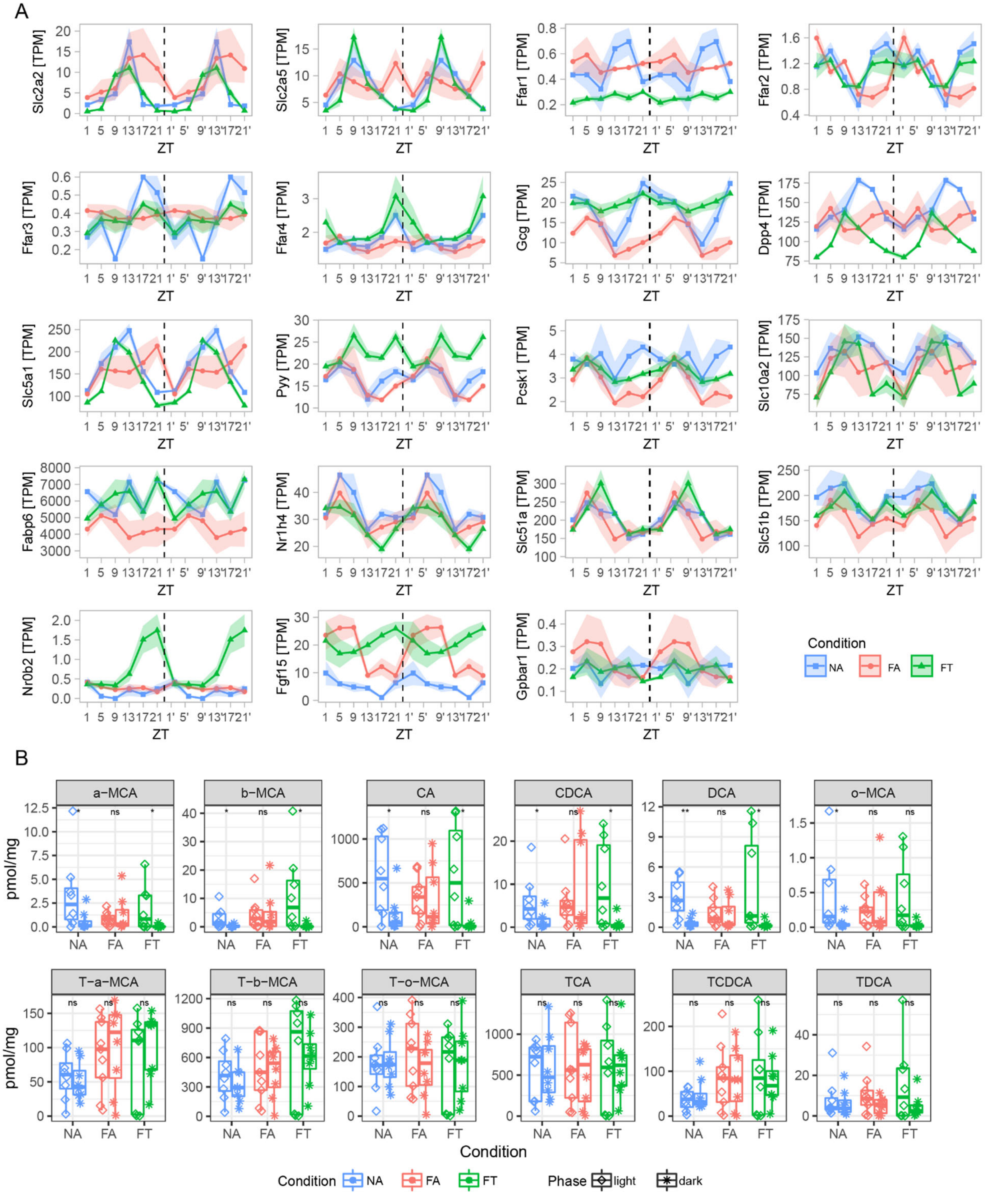

Figure S7. **Levels of gene expression and bile acids, related to Figure 7.**

A) Gene expression of transcripts involved in the GLP-1 and bile acid signaling pathways. Levels are expressed as transcripts per million (TPM).

B) Unconjugated (top) and conjugated (bottom) bile acids levels in ileum samples. Colors represent: NA=blue; FA=red; FT=green.
